# Bone Regeneration Using Bone Morphogenetic Proteins and Various Biomaterial Carriers

**DOI:** 10.3390/ma8041778

**Published:** 2015-04-15

**Authors:** Zeeshan Sheikh, Mohammad Ahmad Javaid, Nader Hamdan, Raheel Hashmi

**Affiliations:** 1 Faculty of Dentistry, University of Toronto, 150 College Street, Toronto, ON M5S 3E2, Canada; 2 Division of Periodontics, Faculty of Dentistry, the Nobel Biocare Oral Health Centre, the University of British Columbia, 2151 Wesbrook Mall, Vancouver, BC V6T 1Z3, Canada; E-Mail: mohammad.javaid2@mail.mcgill.ca; 3 Division of Periodontics, Dental Diagnostic and Surgical Sciences, Faculty of Dentistry, University of Manitoba, D343-790 Bannatyne Avenue, Winnipeg, MB R3E 0W2, Canada; E-Mail: naderful@gmail.com; 4 Department of Emergency and Trauma, South City Hospital, Street 1, Block 3, Sharah-e-Firdous, Clifton Karachi 75400, Pakistan; E-Mail: rhashme@hotmail.com

**Keywords:** bone morphogenetic proteins, BMPs, bone regeneration, biomaterials, carrier methods

## Abstract

Trauma and disease frequently result in fractures or critical sized bone defects and their management at times necessitates bone grafting. The process of bone healing or regeneration involves intricate network of molecules including bone morphogenetic proteins (BMPs). BMPs belong to a larger superfamily of proteins and are very promising and intensively studied for in the enhancement of bone healing. More than 20 types of BMPs have been identified but only a subset of BMPs can induce *de novo* bone formation. Many research groups have shown that BMPs can induce differentiation of mesenchymal stem cells and stem cells into osteogenic cells which are capable of producing bone. This review introduces BMPs and discusses current advances in preclinical and clinical application of utilizing various biomaterial carriers for local delivery of BMPs to enhance bone regeneration.

## 1. Introduction

Trauma, tumor resection, and disease frequently result in fractures or critical sized bone defects. As they do not usually heal spontaneously, their management necessitates bone grafting or major surgical reconstruction [1]. About 5%–10% of all these procedures are associated with impaired healing, which results in psychological stress and morbidity to patients and incur significant economic cost to society. According to the American Academy of Orthopaedic Surgeons more than 6.3 million people suffer from bone fractures annually in the U.S alone [2]. The management of around 25% of those requires some sort of bone grafting [3,4]. Many of these procedures involve the use of autogenous bone that is commonly harvested from the iliac crest [5]. Recent studies have demonstrated that harvesting iliac crest bone grafts is associated with increased risk of morbidity [6,7,8]. It has also been shown that two years following the iliac crest bone graft surgery, up to 25% patients may still feel donor site pain [9]. This potential morbidity together with the limited procurement of autogenous bone has long been the driving force for scientists to develop improved bone graft substitutes [10].

Bone graft substitutes can be broadly categorized into two major types [9]. The first are the osteoconductive materials. These are essentially bone void fillers that allow bone in-growth and are usually made of bioresorbable materials. Common examples include collagen composites, sea-coral, and various ceramics [4,9,11]. Based on the size of defect, these can be used alone or in conjunction with autogenous bone as they are deemed ineffective as a sole treatment for critical sized bone defects [9]. The second category of bone graft substitutes are referred to as the osteoinductive materials, which comprise substitutes that contain biological factors, such as growth factors. These factors can recruit progenitor cells, induce their differentiation into bone forming cells (osteoblasts) and form bone even in a non-osseous site [9]. Because of their tremendous potential to heal and regenerate lost tissue, many osteoinductive materials are currently being investigated for various tissue engineering applications.

Over the years the advent of tissue engineering has been seen as a promising alternate to the current standard of care and can potentially circumvent many limitations encountered with conventional autogenous grafts involving additional surgical procedures [12]. Tissue engineering utilizes the patient’s own precursor cells, matrices, and growth factors to regenerate the lost tissues. Since the early promise shown by research in this field, bone regeneration has received much more interest, as bone is one of the tissues with highest regenerative potential in human body [13]. Bone regeneration can be considered as recapitulation of embryonic bone development because bone heals via generation of new bone instead of scar tissue formation [14]. This process of bone healing or regeneration involves intricate network of molecules including bone morphogenetic proteins (BMPs) [15]. BMPs are the very promising as well as the most intensively studied group of growth factors that are involved in the enhancement of bone healing [16,17,18]. Ever since Urist’s discovery of BMPs as bone inducing proteins, interest in tissue engineering of bone for orthopaedic, craniofacial and periodontal applications has increased exponentially [13,17,19,20,21,22,23,24,25]. Many research groups have shown that BMPs can induce differentiation of mesenchymal stem cells and stem cells into osteogenic cells which are capable of producing bone [26,27,28]. BMPs and their various types are introduced and a discussion of current advances in preclinical and clinical application of various biomaterial carriers for local delivery of BMPs to enhance bone regeneration is presented in this review.

## 2. Structure of BMPs

BMPs belong to a larger superfamily of proteins referred to as transforming growth factors beta (TGF-β) superfamily [15]. The TGF-β superfamily can be broadly categorized into TGFs, BMPs (excluding BMP-I which is a proteinase and a member of tolloid like proteins), growth factors 1–10, which are considered a subclass of BMPs, Vg related genes, glial derived neurotropic factor, inhibins, activins, nodal related genes, and drosphila genes [29]. BMPs are produced as large precursor proteins which undergo disulphide bond dimerization before they are proteolytically cleaved at consensus Arg-X-X-Arg site, yielding mature dimers [30]. Studies have revealed that stability of the processed mature protein is controlled by N-terminal region and efficiency of cleavage is determined by downstream sequence adjacent to cleavage site [26,31]. It has also been suggested that this enzymatic cleavage takes place prior to secretion of BMPs [30]. Following secretion, BMPs can bind to two classes of transmembrane receptors (type 1 and type 2) that are known to have serine threonine kinase activity [30,32,33]. Ligand binding is required for type 1 receptor kinase activation; whereas, activity of type 2 receptor kinase is constitutive. However, optimal ligand binding requires presence of both type 1 and type 2 receptors [30]. Once ligand attaches to type 2 receptor, it transphosphorylates type 1 receptor which leads to activation of type 1 kinase. This, in turn, leads to phosphorylation of members of Smad (protein) family of transcription factors, which are then translocated to the nucleus where subsequent expression of target genes takes place [30,34].

To date, more than 20 types of BMPs have been identified (Table 1) [30,35] but it has been shown that only a subset of BMPs can induce de novo bone formation [14]. Although the mechanism by which BMPs induce osteoblastic differentiation still remains to be elucidated, it is known that these growth factors play a pivotal role in regulation of osteoblastic differentiation [36]. A substantial body of evidence suggests that BMPs like BMP-2, BMP-7, and BMP-9 can provide primordial signal for differentiation of osteoprogenitor cells into osteoblasts which then form the bone extracellular matrix [37,38,39,40,41,42]. Preclinical studies have shown that recombinant human forms of BMPs, especially BMP-2, BMP-4 and BMP-7 can regenerate lost tissue when used with an adequate carrier in critical sized bone defects [36,43,44]. Promising data from these preclinical studies together with encouraging results from clinical trials have found the basis for approval of rhBMP-2 and rhBMP-7 for clinical use by FDA [36,45]. These growth factors have been studied extensively during the last two decades and different recombinant human BMPs (rhBMPs) are currently being investigated for their potential use in several tissue engineered products which may lead to complete regeneration of bone [13,21,46,47,48,49,50,51,52,53,54,55,56,57]. Currently available BMP based applications include BMP loaded synthetic or natural delivery systems and BMP induced differentiation of patient’s transplanted stem cells for later body implantation [13,58,59,60].

**Table 1:** Types, organs of expression and functions of BMPs.

BMP Type	Human Chromosome	Expression in Human Tissues	Functions Performed in Humans
**BMP-1**	8p21.3	Heart, Skeletal Muscle, Kidney, Lung, Liver, Pancreas, Prostate, Bone Marrow, Thymus, Spleen, Spinal Cord, Brain	Metalloprotease that cleaves COOH–propeptides of procollagens I, II, and III/Induces cartilage formation/Cleaves BMP antagonist chordin [61]
**BMP-2**	20p12	Pancreas, Spleen, Kidney, Lung	Skeletal repair and regeneration/Heart formation [62,63]
**BMP-3 (osteogenin)**	4q21.21	Bone Marrow, Spleen, Brain, Heart, Prostate, Pancreas, Skeletal Muscle, Thymus,	Negative regulator of bone Morphogenesis [64]
**BMP-3b (GDF10)**	10q11.22	Spinal Cord, Skeletal, Muscle, Prostate, Brain, Pancreas	Cell differentiation regulation/Skeletal morphogenesis [65]
**BMP-4 (BMP-2b)**	14q22–q23	Brain, Spinal Cord, Spleen, Thymus, Bone Marrow, Heart, Skeletal Muscle, Kidney, Lung, Liver, Prostate, Pancreas	Skeletal repair and regeneration/Kidney formation [66,67,68]
**BMP-5**	6p12.1	Prostate, Spleen, Thymus, Bone Marrow, Brain, Spinal cord, Pancreas, Lung, Heart, Skeletal Muscle, Kidney	Limb development/Bone and cartilage morphogenesis/Connecting soft tissues [69,70]
**BMP-6 (Vrg1, Dvr6)**	6p24–p23	Bone Marrow, Thymus, Heart, Skeletal Muscle, Spleen, Brain, Spinal Cord, Muscle, Kidney, Lung, Liver, Prostate, Pancreas	Cartilage hypertrophy/Bone morphogenesis/Nervous system development [71,72]
**BMP-7 (OP1)**	20q13	Brain, Spinal Cord, Prostate, Thymus, Bone Marrow, Spleen, Heart, Skeletal Muscle, Kidney, Lung, Liver, Pancreas	Skeletal repair and regeneration/Kidney and eye formation/Nervous system development [72,73,74,75]
**BMP-8a (OP2)**	1p34.3	Pancreas, Heart, Kidney, Thymus, Bone Marrow, Spleen, Brain, Spinal Cord, Lung, Prostate	Bone morphogenesis/Spermatogenesis [76]
**BMP-8b**	1p35–p32	Brain, Spinal Cord, Heart, Bone Marrow, Pancreas, Spleen, Skeletal Muscle, Kidney, Liver	Spermatogenesis [76]
**BMP-9 (GDF2)**	10q11.22	Liver	Bone morphogenesis/Development of cholinergic neurons/Glucose metabolism/Anti-angiogenesis [77]
**BMP-10**	2p13.3	Thymus, Spleen, Brain, Spinal Cord, Heart, Lung, Pancreas, Prostate, Bone Marrow, Skeletal Muscle, Liver	Cardiac morphogenesis [77]
**BMP-11 (GDF11)**	12q13.2	Thymus, Bone Marrow, Pancreas, Spinal Cord, Brain, Spleen	Patterning mesodermal and Neural tissues, Dentin Formation [78]
**BMP-12 (GDF7/CDMP2)**	2p24.1	Data not available	Ligament and Tendon development/Sensory neuron development [79]
**BMP-13 (GDF6/CDMP2)**	8q22.1	Data not available	Normal formation of bones and joins/Skeletal morphogenesis/Chondrogenesis [80,81]
**BMP-14 (GDF5/CDMP1)**	20q11.2	Bone Marrow, Kidney, Liver, Heart	Skeletal repair and regeneration [75]
**BMP-15 (GDF9b)**	Xp11.2	Data not available	Oocyte and Follicular development [82]
**BMP-16**	Data not available	Data not available	Skeletal repair and regeneration [83]
**BMP-17**	Data not available	Data not available	Data not found [84]
**BMP-18**	Data not available	Data not available	Data not found [84]

Biological function, structure, physiology, signaling pathways and regulation of BMPs expression have already been reviewed in detail elsewhere [15,26,29,30,85,86]. In the present review we discuss the promising results obtained from the use of BMPs based tissue engineered bone constructs from preclinical experiments as well as clinical studies and the significant strides that have been made in recent years in the field of BMPs carriers.

## 3. Carriers for BMPs

Although it has been shown that BMPs can initiate bone formation in solution form [87], the dose required to induce bone formation can be dramatically reduced if BMPs are implanted with an appropriate carrier [87]. The principle role of BMP carrier system is to retain these growth factors at the site of injury for prolonged period of time and render an initial support for cells to attach and elaborate the extracellular matrix with subsequent regeneration of lost architecture [13]. There are a variety of biomaterials that can and have been investigated for BMP delivery for bone tissue engineering applications (Table 2). A carrier should ideally induce optimal inflammatory response, should be completely biodegradable and present adequate porosity for infiltration and proliferation of cells and sprouting blood vessels at the site of new bone formation [13]. Moreover, it should prevent degradation of BMPs while maintaining their bioactivity and allow a sustained release in a controlled manner to promote new bone formation at the site of defect [13,18,88,89,90]. Lastly, it should be easily sterilized, easy to handle and stable when stored and be commercially feasible allowing scale up production. In this section we discuss various carriers that have shown great promise and can potentially be used to construct an efficient and effective BMPs based tissue engineered bone construct.

**Table 2:** Biomaterial carriers investigated for BMP delivery in bone tissue engineering.

Carrier	BMP	Matrix type	Model
**Synthetic Polymers**			
**PGA**	BMP-2	Membrane	Dog periodontal repair [91]
	BMP-2	Scaffolds	Cervical spinal fusion in goats [92]
	BMP-9	Scaffolds	*In vitro* bone/cartilage formation [93]
**PLGA**	BMP-2	Scaffolds	Orbital floor reconstruction in sheep [94]
	BMP-2	Scaffolds	Rabbit femoral head necrosis [95]
	BMP-2	Scaffolds	*In vitro* release and rat calvaria [96]
	BMP-2	Scaffolds	Canine mandibular defects [97]
	BMP-2	Scaffolds	Alveolar ridge defects in rats [98]
	BMP-2	Scaffolds	Rabbit radius defects [99]
	BMP-2	Scaffolds	Alveolar cleft repair in dogs [100]
**PLGA-gelatine**	BMP-2	Composites	Rabbit ulnar defects [101]
	BMP-2	Hydrogel/scaffold	Rabbit knee cartilage/bone interface [102]
	BMP-2	Composites	Dog tooth defects [103]
	BMP-2	Composites	Dog tibia defects [101]
**PLGA-fibrin**	BMP-2	Sealant	Rabbit radial bone defect [104]
**PLGA-heparin**	BMP-2	Composites	Ectopic model in rat [105]
**PLA**	BMP-2	Scaffolds	Rabbit ulna [106]
	BMP-2	Composite	Radial defects in rabbit [107]
	BMP-2	Scaffolds	Ectopic bone formation in rats [108]
**PLA-collagen**	BMP-2	Membrane	Ectopic bone formation in rabbits [109]
	BMP-2	Composite	Rat ectopic bone formation [110]
**PLA-DX-PEG**	BMP-2	Scaffolds	Femoral canine model [111]
	BMP-2	Scaffolds	Rat cranial defects [112]
	BMP-2	Scaffolds	Mice ectopic bone formation [113]
**PEG-based**	BMP-2	Hydrogels	Rat cranial defects [114,115]
	BMP-2	Hydrogels	Rat critical sized cranial defects [116]
	Bmp-2	Hydrogels	*In vitro* release profiling [117]
**PEG-based, heparin**	BMP-2	Hydrogels	Rat critical sized calvarial defects [118]
**Isopropylacrylamide**	BMP-2	Hydrogels	Ectopic bone formation [119]
**Natural Polymers**			
**Fibrin**	BMP-2	Gels	Rabbits, dogs, rats and cats; various bone defects [120,121,122]
	BMP-2	Sealant	Ectopic bone formation in mice [123,124]
	BMP-2	Sealant	Human frontal bone defect [125]
	BMP-2	Sealant	Differentiation of rabbit bone marrow cells [126]
**Fibrin-collagen**	BMP-2	Sealant in sponge	Rat spinal model [127]
**Fibrin-heparin-collagen**	BMP-2	Sponge	Mouse calvarial defects [128]
**Fibrin-heparin**	BMP-2	Sponge	Spinal fusion in rabbit [129]
	BMP-2	Sponge	Posterior lumbar fusion in rabbits [130]
**Gelatine**	BMP-2	Hydrogel	Rabbit skull defects [131]
	BMP-2	Hydrogel	Non-human primate skulls [132]
	BMP-2	Hydrogel	Ectopic bone formation in mice [133,134]
**Hyaluronic acid**	BMP-2	Hydrogel	Ectopic bone formation in rats [135]
	BMP-2	Hydrogel	*In vitro* release model [136]
	BMP-2 & 4	Sponges	Rat mandibular defects [137,138]
	BMP-2	Sponges	Dog alveolar ridge defects [139]
	BMP-2	Scaffolds	Periodontal repair in dogs [91]
	BMP-2	Gels	Osteotomy in non-human primates [140]
	BMP-2	Gels	Non-union tibial defects in rabbits [141]
**Hyaluronic acid -PLA**	BMP-2	Composite	Rat femurs critical sized defects [142]
**Silk fibroin**	BMP-2	Films	Cranial defects in mice [143,144]
	BMP-2	Nano-fibers (electrospun)	Differentiation of human bone marrow cells [145]
	BMP-2	Scaffolds	Critical sized defects in rats [143]
	BMP-2	Scaffolds	Cranial defects in mice [146]
**Alginate**	BMP-2	Hydrogels	Ectopic bone formation in mice [147]
	BMP-2	Gels	Tibial defects in rats and ectopic bone formation [112,148,149]
	BMP-2	Gels	Rabbit radial bone defects [150]
**Chitosan**	BMP-2	films	C2C12 cell line differentiation [151]
	BMP-2	Membranes	Osteoblast cell differentiation [152]
**Chitosan-collagen**	BMP-7	Scaffold	Cell differentiation [153]
**Chitosan-alginate**	BMP-2	Gel	Mice trabecular bone formation [154]
**Chitosan-gelatine**	BMP-2	Composite	Osteoblast differentiation [155]
**Dextran**	BMP-2	Hydrogel	Rat ectopic model [156]
**Titanium**			
**Titanium**	BMP-2	Implant (porous)	Dog humerus [157]
	BMP-2	Implant (porous)	Dog mandible [158]
	BMP-7	Implant (threaded)	Rabbit femur [159]
	BMP-2	Particles	*In vitro* assay [160]
	BMP-2	Shell capsule composite	Alveolar bone reconstruction [161]
**Titanium-HA**	BMP-2	Cylinder	Sheep tibia [162]
	BMP-2	Implant coating	*In vitro* evaluation [163]
**Titanium-HA-heparin**	BMP-2	Composite	*In vitro* and *in vivo* (distal femur of rabbit) [164]
**Titanium-chitosan**	BMP-2	Composite	*In vitro* model [165]
**Micro and Nanoscale Carriers & Polymer-Ceramic Composites**			
**PLGA**	BMP-7	Microparticles	Sheep vertebrae [166]
	BMP-2	Microparticles	Rabbit calvarial bone defects [167]
	BMP-2	Microparticles	Osteoblast differentiation *in vitro* [168]
	BMP-2	Microparticles	Rat femurs [169]
	BMP-2	Microparticles	Rat calvarial bone defects [170]
**PLGA-CaP**	BMP-2	Microparticles	Rat cranial and ectopic model [171,172]
**PLA**	BMP-2	Microparticles	Ectopic bone formation in rats [173]
**PLA-PCL**	BMP-2	Nanoparticles	Radius of rabbits [174]
**Collagen-HA**	BMP-4	Microparticles	Rabbit femoral bone defects [175]
	BMP-2	Scaffold	Implantation in rat hind limb [176]
	BMP-2	Scaffold	*In vitro* release study [177]
	BMP-2	Nanocrystals/fibres	Spinal fusion, tibial fractures in dogs [178]
**Dextran**	BMP-2	Nanoparticles	*In vitro* differentiation of rabbit bone marrow cells [179]
**Dextran-PEG**	BMP-2	Microparticles	*In vitro* differentiation of rabbit bone marrow cells [180]
**Dextran-gelatin**	BMP-2	Microparticles	Periodontal regeneration in dogs [181]
**Chitosan-alginate**	BMP-2	Microparticles	Canine defects [182]
**Hyaluronic acid-HA**	BMP-2	Composite	Osteointegration in sheep cancellous bone [183]
**PLA-collagen-HA**	BMP-2	Composite	Mice ectopic bone formation [184]
	BMP-2	Composite	Radius defects in dogs [185]
**PLA-PEG-HA**	BMP-2	Composite	Rabbit radius model [186]
**PLA-DX-PEG-CaP**	BMP-2	Composite	Femur defects in rabbits [187]
	BMP-2	Composite	Femur defects in rabbits [188]
		Composite	Spinal fusion in rabbits [189]
**Fibrin-CaP**	BMP-2	Sealant	Rat calvarial defects [190]
**CaP**	BMP-2	Scaffold (porous)	Maxillary sinus floor elevation in rabbits [191]
	BMP-2	Solid free form fabricated scaffold	*In vitro* and *in vivo* evaluation [192]
**Biphasic CaP**	BMP-7	Scaffold	Ectopic mouse model [193]
**HA-TCP**	BMP-2	Scaffold	Rabbit calvarium [194]
	BMP-2	Scaffold	Ectopic bone formation in rats [195]

Notes: PGA: Poly-glycolic acid; PLGA: Poly-lactic-glycolic acid; PLA: Poly-lactic-acid; DX: Dioxanone; PEG: Poly-ethylene-glycol; HA: Hydroxylapatite; Ca-P: Calcium phosphate; PCL: Polycaprolactone.

### 3.1. Ceramics

Research has shown that ceramics such as hydroxyapatite and other types of calcium phosphate materials can promote formation of bone like mineral surface leading to increased interface between bone and the implanted material [196]. Hydroxyapatite (HA), which comprises about 70% of bone, is an osteoconductive [197,198] material that can be formulated as blocks, disks, powder or granules [199]. Various research groups have tested HA alone [200] or in combination with other polymers for delivery of BMPs [194,201,202]. These studies demonstrate that HA is a very promising carrier for delivery of BMPs not only because it is osteoconductive and aids in retention of growth factors but also because it enhances the delivery of growth factors [47,203,204,205]. HA has very low biodegradation and that is a major disadvantage [196,206]. This limits the amount of newly formed bone that can replace the resorbing graft tissue [206]. To overcome this problem, β-TCP can be added to HA, to create a biphasic calcium phosphate composite material [207]. This has higher resorption rate and well described bioactivity [208,209].

Being osteoconductive and biocompatible, calcium phosphate based ceramics, cements and coatings have also been studied extensively. Association of BMP into a bone-like calcium phosphate could possibly help to control the release of BMP [210]. A major advantage in using calcium phosphate as rhBMPs carrier in comparison to other materials lies in the fact that high doses of rhBMPs are not required for bone formation [13,47,211]. Various studies have shown that rhBMP-2 when delivered through calcium phosphate based delivery systems results in accelerated bone healing [212,213]. Similarly studies on non-human primates have also yielded promising results [214,215]. Calcium phosphate based BMPs delivery systems have tremendous potential for tissue engineering based bone constructs but clinical trials need to be carried out to determine their effectiveness before they can be routinely used as an alternate to autogenous bone grafting procedures.

### 3.2. Non-Ceramics

#### 3.2.1. Synthetic Biodegradable Polymers

Various synthetic polymers have been used extensively in tissue engineering applications [13,216,217,218]. The possibility of prevention of disease transmission in grafting procedures through use of synthetic polymers instigated the scientists to develop synthetic polymer based BMP carriers. Initially Polylactic acid (PLA) due to its adsorptive stability was investigated as a potential BMP carrier in the early 1990s [219], but was soon considered ineffective due to release of acidic degradation by products [220]. Further research lead to development of a new generation of PLA-based synthetic poylmers, including polylactic acid-p-dioxanone-polyethylene glycol (PLA-DX-PEG) and polylactic acide-polyehylene glycol (PLA-PEG) [216,221,222]. Due to its versatile temperature dependent liquid-semi solid transition, PLA-PEG allows percutaneous injection after heating [13]. This injectable approach provides a less invasive alternative to open surgical procedure [218]. Similarly experiments with PLA-DX-PEG showed promising results. It was observed that synchronization existed between new bone formation by BMP and polymer biodegradation [223]. PLA-DX-PEG has been tested by different research groups in various animal models [59,187,222,224]. Further research has tested composites of PLA-DX-PEG with calcium phosphate and demonstrated that combination of calcium phosphate with PLA-DX-PEG reduces the requirement of rhBMP needed to induce new bone formation [13,188,225]. Recently developed composites of hydroxyapatite with PLA-PEG, hydroxyapatite with polyamide and hydroxyapatite with collagen composites have also shown great promise when used in conjunction with rhBMP-2 for tissue repair in different animal models [186,226,227].

Polyglycolic acid (PGA) which has superior mechanical strength when combined with PLA results in Polylactic-co-glycolic acid (PLGA) which has received much attention for tissue engineering applications [228]. Biodegradation of the composite can be controlled by changing the proportion of the two materials [229,230]. PLGA has been tested in various studies and the promising results show tremendous potential of PLGA as a carrier for BMPs [99,100,101,103]. Interestingly when rhBMP was used in conjunction with PLGA, much higher bone formation was observed in comparison to PLGA alone, highlighting the osteoinductive potential of BMPs [94,97,98]. More recently a new approach involving conjugation of heparin to PLGA scaffold was tested by Jeon and co-workers [105]. They reported that the resultant composite demonstrated a much longer sustained release of rhBMP-2, resulting in significantly more new bone formation [105].

As hydrogels contain large amounts of water, they have long been considered potential candidates for proteins and drug delivery [231,232,233,234,235] and many synthetic polymers have already been formulated as hydrogels for BMPs delivery. PEG based hydrogel with extracellular matrix-like characteristics, such as integrin binding sites for cellular attachment and substrates for matrix metalloproteinases (MMPs) for delivery of rhBMP-2 has been reported [114,115]. These studies showed promising results with initial cellular penetration followed by bone tissue formation within the hydrogel [114,115]. In a similar study, Pratt and co-workers [118] demonstrated cellular penetration of PEG-based hydrogel, which was conjugated with heparin and plasmin for improving rhBMP-2 stability [118]. Similarly Fisher and colleagues reported successful use of poly-propylene fumarate-co-ethylene glycol based rhBMP-7 carrying thermo-reversible hydrogel for tissue engineering of cartilage [236]. The authors concluded that these hydrogels could be potentially used for regeneration of cartilage tissue [236]. In another study, Gao and Uludag [119] demonstrated that N-isopropylacrylamide and N-acryloxysuccinimide based hydrogels could be successfully used for effective and controlled delivery of proteins such as BMPs [119]. The main drawback of using synthetic polymers is the risk of potential inflammatory response due to acidic by-products because of polymer degradation [228] which may interfere with the stability of adsorbed BMPs. This has incited scientists to look for other materials that can serve as BMP delivery carriers without such limitations.

#### 3.2.2. Natural Polymers

Ideally an implant based on the principles of tissue engineering should mimic natural environment of tissues and in this context natural polymers can render the additional benefit of accelerated healing as they can send signals to guide cells in various stages of their development [13,176,237]. Various natural polymers including collagen, silk, alginate, agarose, chitin and chitosan have been tested as potential carriers for delivery of BMPs [238]. Many of these materials are developed from substances naturally present in extracellular matrix, cartilage and bone. Therefore, it is no surprise that these materials exhibit excellent properties for potential use in regenerative medicine [239,240].

In a series of studies, Saito and co-workers developed alginate gels incorporated with peptides corresponding to BMP-2 region that binds to cell receptors. Using this gel they demonstrated successful repair of bone defects in rat and rabbit models [149,150,241]. Simmion and co-workers [147] also reported successful delivery of rhBMP-2 in rats using alginate hydrogels [147]. Chitosan derived from alkaline deacetylation of chitin has also been formulated in different forms including fibre meshes [242] and hydgrogels [243] which have shown great promise for use in tissue engineering of bone and cartilage making it a potential candidate for delivery of BMPs [13,244]. In a study by Park *et al.* [154], the authors demonstrated that a composite gel comprising of chitosan and alginate loaded with rhBMP-2 and mesenchymal stem cells could induce new bone formation [154]. In another *in vitro* study, Liang and co-workers observed that when rhBMP-2 was incorporated in a chitosan-gelatin based scaffold, it increased the expression of osteocalcin, a biomarker of osteoblast cell lines [155]. It has been shown that chitosan and PGA, and chitosan and collagen based composite scaffold for delivery of rhBMP-2 has tremendous potential in bone regenerative therapies due to enhanced release amount and sustained release of rhBMP-2 [153,245].

Fibrin, which can be formulated in an adhesive glue like delivery system [246] has also been studied as a potential carrier for BMPs delivery in different animal models. It has been tested *in vivo* as a carrier for rhBMP-4 [247] and rhBMP-2 [190] in the form of fibrin-fibronectin sealing system and for rhBMP-2 [248] in the form of fibrin sealant. These studies demonstrated much higher bone formation in test sites where fibrin carrier was loaded with rhBMP as compared to control sites. Other research groups have also reported development of fibrin matrices for delivery of rhBMPs [120,121,122]. These matrices were used to treat bone defects in rat, cats and dogs. They reported bridging of the bone defect with functional bone healing, demonstrating effectiveness of this delivery system. All in all, fibrin-based BMPs carriers are a valuable addition to bone engineering scaffolds considering they promote bone formation [249] and allow retention of growth factors [250].

Hyaluronans distributed widely throughout the connective tissue can also been formulated into pads, sponges and gels for delivery of rhBMPs. In a study by Kim and Valentini [251], the authors demonstrated that hyaluronan based rhBMP-2 carrier retained higher concentration of BMP in comparison to collagen gels [251]. Since then hyaluronan based carriers have been used in a number of studies to deliver rhBMP [139,140,141,142,183,252]. More recently hyaluronic acid based carrier was used to deliver BMPs for treatment of mandibular defects in rats. The authors found that significantly more bone was formed when rhBMP-2 was used in addition to carrier in comparison to carrier alone [137,138].

Silk has also been suggested as a possible carrier for BMPs delivery. Derived from silkworm larvae cocoons, silk has been extensively investigated by various research groups for use as a BMPs vehicle [13]. Following *in vitro* and *in vivo* studies, Karageorgiou and colleagues reported that rhBMP-2 retained its activity when it was directly immobilized on silk fibroin films [144]. In another study, silk-based scaffold was used to deliver rhBMP-2 [145]. The authors reported that this delivery system induced osteogenesis in cultures of mesenchymal stem cells with increase in alkaline phosphatase activity and calcium deposition [145]. Similar results were obtained when silk fibroin scaffold was used to deliver BMP-2 at bone defect sites in mice [143]. Others have also reported promising results when rhBMP-2 in combination with human mesenchymal stem cells delivered through silk fibroin scaffold were used in treatment of critical sized bone defects in rats [146]. In comparison to other protein-based materials, degradation rate of silk is slower which allows sufficient time for bone healing. This particular advantage makes silk a very promising candidate for delivery of BMPs and for development of various bone tissue engineering constructs [253].

Collagen is the most abundant protein in mammalian connective tissue and is the main non-mineral component of bone. It can also positively influence cellular infiltration and wound healing. Another advantage lies in its ability to be processed in aqueous form [13]. Furthermore collagen and its breakdown products are also physiologically biocompatible [13,153] and, hence, it is no surprise that many collagenous formulations including demineralised bone matrix, collagen strips, resorbable collagen sponges, collagen gels, and fibril collagen have been prepared for applications in tissue engineering [51,153,176,254,255,256,257].

Versatility, wettability and ease of manipulation has led many scientists to test the possibility of use of collagen sponges as a carrier for delivery of rhBMPs in various tissue engineering applications including fractures and critical sized bone defects [176,258]. Numerous studies have revealed safety and effectiveness of collagen sponge and two collagen sponge delivery systems have been approved by FDA for delivery of rhBMP-2 and rhBMP-7 as an alternate to bone grafts for spinal fusion and long bone fractures [258,259,260]. However, the collagen used in these carriers is of animal origin and poses a risk of immunological reaction and possibility of transmission of infectious agents and diseases [261,262] and hence scientists are constantly striving to develop a superior delivery system for BMPs.

#### 3.2.3. Titanium

BMPs were first tested in 1994 for surgical reconstructions in craniomaxillofacial surgery using titanium implants [263]. Titanium implants treated with BMP-2 [264] have also been tested along with bioactive titanium dioxide/hydroxyapatite surfaces functionalized with BMP-2 *in vitro* [163,265]. *In vivo* testing of bone response to titanium implants with BMP has also been evaluated [266]. In a sheep model, the osteointegration of hydroxyapatite-titanium implants coated with non-glycosylated BMP-2 was evaluated and showed promising bone response [162]. Osteoblast differentiation and mineralization promoted by a globular fibrinogen layer through cell autonomous BMP signaling on titanium carrier surfaces has been studied [54]. Greater bone formation was demonstrated on apatite-coated titanium with incorporated BMP-2/heparin *in vivo* [267]. The effect of immobilization of heparin and BMP-2 to titanium surfaces has been studied for improving osteoblast function and osteointegration [169,268,269,270,271]. Surface modification of titanium with hydroxyapatite-heparin-BMP-2 has been shown to enhance the efficacy of bone formation and osseointegration *in vitro* and *in vivo* [164]. Fabrication of printed titanium shells for containment of BMP-2 composite graft materials for alveolar bone reconstruction has also been researched upon [161].

### 3.3. Microspheres, Nanoparticles and Ceramic/Polymeric Composite Microspheres

Over the years great deals of resources have been invested in the area of micro and nanoparticles in search of simple, efficient and cheap drug delivery systems. Researchers have also tested microspheres and nanoparticles for delivery of BMPs [13]. Following promising results of PLGA based BMPs delivery systems, microshperes of PLGA have been studied in various animal models including calvarial bone defects in rats [170], rat femur [272] and rabbit calvarial defects [167]. These studies demonstrated that presence of rhBMP within PLGA microspheres was necessary for bone formation [272] and resulted in restoration of normal contouring and radiopacity of defects whereas only soft tissue formation was observed when PLGA microshperes were used alone [170].

Wang and colleagues have also evaluated collagen-hydroxyapatite composite microspheres for delivery of BMPs. They observed that when BMP-4 based particles were implanted in rabbit femoral defects, significant bone formation took place in comparison to influx of inflammatory cells and fibrous tissue formation at sites, which were treated with carrier particles alone [175]. Recently Chen and co-workers carried out a series of interesting studies where they used dextran based microshperes and nanoparticles for delivery of BMPs [273,274]. They reported that by varying proportion of the constituent components, the release of rhBMP could be increased to more than 28 days [275]. Novel approaches that use nanoparticles of sulphated chitosan, hydroxylapatite, silica, metallofulerene have also been explored to deliver BMPs for bone tissue engineering applications [53,57,276,277,278,279,280,281,282,283]. Although there are some unresolved issues in use of microspheres or nanoparticles for delivery of BMPs like inadequate mechanical strength of scaffold or loss of bioactivity of growth factor [284] but nanoparticle technology is one of the most promising approaches for future of tissue engineering of bone.

## 4. Bone Regeneration Using BMPs

### 4.1. Preclinical Studies

Research has revealed that BMPs play a critical role in growth and differentiation of various cell lines including osteoblasts [15]. A number of preclinical experiments including animal studies have demonstrated the effectiveness of recombinant human BMPs in regeneration of bone [27,285,286,287,288,289,290,291,292,293,294]. Many of these preclinical studies used critical sized bone defect model. In bone, “*critical sized defects*” are defined as defects that do not heal without intervention [295]. For instance healing of critical sized bone defects by BMP-2 was shown in different species including rabbits, sheep, dogs and non-human primates [17,85]. Healing of bone defects using genetic approach where an implant comprising of a bioresorbable polymer mixed with mesenchymal stem cells transfected with adenovirus BMP-2 has also been reported [85]. It has also been shown that systemic administration of rhBMP-2 results in increased activity of mesenchymal stem cells and reversal of age related and ovariectomy induced bone loss [296]. Recently different research groups have also shown that rhBMP-2 when delivered on a calcium phosphate carrier or with liposome carrier, results in accelerated bone healing in rat and rabbit models [85]. In another study complete bone regeneration was observed when rhBMP-2 soaked collagen was grafted in critical sized calvarial defects in rats [297]. Similar results were observed by Yasko and co-workers following grafting of rhBMP-2 in rat femoral defects [298]. In another study, femoral defects in sheep showed evidence of new bone formation four weeks post rhBMP-2 grafting. Eight weeks later complete bone union was verified by radiographical analysis. Histological evaluation after 52 weeks of implantation revealed presence of woven and lamellar bone [299]. A study in dogs evaluating the role of rhBMP-2 in bone defects revealed complete healing of mandibular defects within three months. The authors then assessed the bone quality by degree of mineralization, bone thickness and biomechanical strength over the three months. They observed significant improvement in all three parameters [300]. In a series of recent studies, Cook and colleagues demonstrated that grafting of collagen based rhBMP-7 particles resulted in restoration of critical sized bone defects in rabbits and dogs. Radiographical evidence of complete union was observed at the end of two months. Biomechanical experiments showed that mean torsional strength of the unions was comparable to that observed in intact bone [285,301]. In a study in non-human primates by Ripamonti and co-workers, evidence of new bone formation was observed as early as 15 days post-surgery and complete bone formation was achieved in three months [302]. Similar results were obtained when rhBMP-7 was grafted in sinuses and dental extraction sites in chimpanzees [303]. All these studies suggest that BMPs can lead to complete healing of critical sized bone defects in short period of time in various species.

### 4.2. Clinical Studies

It is surprising that despite the positive role BMPs play in accelerating fracture repair and bone healing [18,22,304,305,306,307,308], they have been studied only to a limited extent in human clinical trials. However, in the last decade many clinical studies were conducted which yielded promising and encouraging as summarized in (Table 3).

**Table 3:** Clinical studies carried out using BMPs for bone tissue engineering.

Clinical Studies Using BMP-2				
Authors		Type of Fracture	Methods	Findings
Herford, A.S. and Boyne, P.J. [309]		Mandibular Continuity Defect	Patients were treated with rhBMP-2 alone or in conjunction with collagen carrier without concomitant bone material.	Successful osseous restoration of critical sized edentulous area was observed which was then followed by prosthetic treatment.
Sweeny, L., Lancaster, W.P., Dean, N.R., Magnuson, J.S., Carroll, W.R., Louis, P.J., Rosenthal, E.L. [310]		Mandible	Test Group: Standard treatment plus rhBMP-2.	There was no significant difference in the measured outcomes between the two groups.
			Control Group: Standard treatment without use of rhBMP-7.	
Govender, S., *et al* [311]		Open Tibial Shaft Fractures	Control Group: Received standard of care.	The implant containing 1.5 mg/mL rhBMP-2 was significantly superior to standard of care in accelerating fracture and wound healing, reducing of rate of infections and frequency of secondary interventions. It also reduced the overall invasiveness of the procedure.
			Test Group: Received standard of care with implant containing rhBMP-2 in concentration of 0.75 mg/mL or 1.5 mg/mL.	
Tressler, M.A., Richards, J.E., Sofianos, D., Comrie, F.K., Kregor, P.J., Obremskey, W.T. [312]		Long Bone Non-unions	Patients were given standard treatment with iliac crest bone graft or rhBMP-2.	No statistically significant difference was observed in rate of healing and postoperative infection. Iliac bone graft resulted in significantly more intraoperative blood loss and longer operative procedures.
Bibbo, C., Patel, D.V., Haskell, M.D. [313]		High risk ankle and hind foot fusion	Patients were treated with standard of care in conjunction with rhBMP-2.	Successful union was achieved in 96% fracture sites. The authors concluded that rhBMP-2 is an effective adjunct for treatment of high risk ankle and hind foot fusions.
**Clinical Studies Using BMP-7**				
**Authors**	**Type of Fracture**		**Methods**	**Findings**
Moghaddam, A., Elleser, C., Biglari, B., Wentzensen, A., Zimmermann, G. [314]	Long Bone Non-unions		Patients who had atrophic non-union of long bones were treated with rhBMP-7 in a Type 1 Collagen carrier without concomitant bone graft material, with bone graft and bone graft and osteosynthesis revision.	Successful union was observed in 82% fractures. Healing was confirmed clinically as well as radiographically.
Dohin, B., Dahan-Oliel, N., Fassier, F., Hamdy, R. [315]	Persistent non-union involving different bones		OP-1 (BMP-7) with Type 1 Collagen carrier was used in conjunction with standard of care.	Clinical and radiographical evidence of bone healing was observed in 74% patients. The authors concluded that OP-1 stimulates healing of persistent non-union without serious adverse effects.
Moghaddam-Alvandi, A., Zimmermann, G., Büchler, A., Elleser, C., Biglari, B., Grützner, P.A., Wölfl, C.G. [316]	Non-union in Long Bones		rhBMP-7 was applied in non-union fracture of long bones. Before application of rhBMP-7 patients had already underwent surgical treatment an average of 3.3 times.	Proper bone healing was observed in 92% fracture sites. The authors concluded that although rhBMP-7 may not be used in all non-union cases, it appears to be effective in treatment of complex cases.
Nicodemo, A., Capella, M., Deregibus, M., Massè, A. [317]	Non-union Sacral Fracture		Patients who had previously failed to respond to standard of care were treated with rhBMP-7 as an adjunct to standard of care.	The use of rhBMP-7 resulted in successful healing of fractures which had previously failed to heal with traditional surgical techniques.

A study by Govender and co-workers [311] showed that when rhBMP-2 was delivered using an absorbable collagen sponge for treatment of open tibial fractures, there was a 44% reduction in risk of failure of healing. The authors also reported significantly less secondary invasive interventions and overall shorter healing time in comparison to control group [311]. This combination of rhBMP-2 and absorbable collagen has been approved by Food and Drug Administration (FDA) and regulation authorities in Europe and is being commercialized under the name of InFuse in the US and InductOs in Europe. In a similar study, rhBMP-7 bound to bovine type 1 collagen was compared to autogenous bone graft for treatment of non-union tibial fractures. Although there was no improved healing with rhBMP-7 but the study showed that results with rhBMP-7 and bovine type 1 collagen were comparable to autogenous bone graft, which is considered the gold standard in treatment of critical sized bone defects [318]. In another study by Canadian Orthopaedic Trauma Society, the use of rhBMP-7 for treatment of open tibial shaft fractures was evaluated. Patients were randomly divided into test groups which received rhBMP-7 and control groups where rhBMP-7 was not part of the treatment modality. Clinical, radiological and serological testing revealed that a significantly larger number of patients in the test group were able to fully bear weight without pain at the 12 month follow up period in comparison to the control group. Secondary intervention for delayed union and non-union was also significantly lower in the rhBMP-7 group as compared to the control group. Furthermore, no rhBMP-7 related adverse effects were encountered [319].

In a similar study, use of rhBMP-7 was evaluated for treatment of distal tibial fractures [320]. Patients treated with hybrid external fixation and BMP-7 was compared with patients who received similar treatment without the use of rhBMP-7. Authors reported that the mean time for removal of external fixator, mean time to achieve union and mean time off work were significantly lower in the rhBMP-7 treated group compared to the control group [320]. BMP-7 has also been evaluated in a prospective randomized controlled trial for treatment of proximal pole scaphoid non-unions [321]. The patients were divided into three groups: Group 1 received autografts alone, Group 2 received autografts with rhBMP-7, and Group 3 received allograft with rhBMP-7. Clinical, radiological and scintigraphic assessment revealed that rhBMP-7 improved performance of both autografts and allografts. Radiological evidence of healing in patients treated with autografts and rhBMP-7 was four weeks in comparison to nine weeks for patients treated with autografts alone. Furthermore clinical outcome of patients treated with allograft and rhBMP-7 was equal to Group 1 where patients were treated with autografts alone [321]. In a prospective study, twenty-three consecutive patients with atrophic humeral diaphyseal non-unions were treated with compression plates or intramedullary nails in conjunction with rhBMP-7. The authors reported that union was achieved in all patients without any serious complications or adverse effects. They concluded that rhBMP-7 was safe and effective for treatment of humeral diaphyseal non-unions [322].

In a recent prospective single arm study [323], McKee and colleagues examined the effectiveness of rhBMP-7 for treatment of atrophic long bone non-unions. Participating patients on an average had 2.1 previous operations and autogenous iliac crest bone grafting had already failed in 28 of them. All the patients underwent revision fixation with application of rhBMP-7 at the non-union site. The authors reported that 54 of the original 61 non-union cases had healed at the conclusion of the study and that there were no anaphylactic reactions or adverse effects associated with the use of rhBMP-7 [323]. In another study, a case review was made of 14 patients who underwent treatment for lesions of the body and angle of the mandible resulting from neoplasms or osteomyelitis. The patients were treated with rhBMP-2 on a collagen carrier without concomitant use of bone materials. The study revealed that all the cases had successful osseous restoration of the edentulous area, which was subsequently followed by prosthetic treatment. The authors concluded that use of rhBMP-2 in treatment of critical sized mandibular defects without concomitant use of bone grafts resulted in excellent regeneration of affected area allowing the restoration of prosthodontic function [309].

## 5. Controversy with rhBMPs

Although BMPs are being studied extensively for bone tissue engineering and repair applications, controversy exists. This is regarding the success and failure of rhBMP-2 in conflicting reports. The muddled reporting of its clinical superiority to autografts from iliac crests and the failure to report or underreporting of adverse side effects from its use exist [15,16,324,325]. Since 2006, independent research studies started demonstrating 20%–70% failure rates with the use of rhBMP-2 [326]. Seroma formation, bone over growth, retrograde ejaculation and increased risk of neoplastic changes are the most common complications associated with their use. The FDA placed a warning on BMP use in June 1998 in cervical spine applications due to extreme postoperative dysphagia [325,327]. The *Wall Street Journal* reported that Medtronic was under investigation for off label use of INFUSE (rhBMP-2) [327]. Allegations came forward of cherry picked research results and fraud by the author in a study showing the effective use of rhBMP-2 [328]. It was revealed that the author had a conflict of interest and financial ties to the manufacturer of rhBMP-2 [327]. This led to the reputation of rhBMP-2 being tarnished and it clinical usefulness questioned. The question that was being raised was that is the advantage gained by the use of BMP worth the risks it poses. In order to clear some of the confusion, systemic reviews and meta-analysis of results were conducted independently by Yale. The reports found that the current data in whole does not show a significant improvement in fusion rates with the use of rhBMP-2 as compared to autograft iliac crest bone graft used alone [325,326,327]. Both BMP-2 and iliac crest bone graft were shown to be associated with similar rates of neurological and retrograde ejaculation and complications when used in posterolateral or anterior interbody lumbar fusion [325,327]. It was concluded that BMP-2 use results higher rates of ectopic bone formation in posterior lumbar interbody procedures and high rates of complication in anterior cervical procedures [326,327]. Although there is a slight risk of cancer with the use of BMP-2, the absolute risk remains extremely small and, therefore, clinically insignificant [18,327].

## 6. Future Challenges

The discovery of BMPs ushered a new era not only in understanding of bone physiology but also in development of new methods for treatment of defects that require orthopaedic and maxillofacial surgery [13,24]. During the last decade, many successful preclinical and clinical studies have been carried out which are a testament to the tremendous potential of BMPs for use in tissue engineering applications for treatment of bone defects. These cytokines have the unique capacity to initiates bone formation not only in osseous tissues but also in extra-skeletal sites [14,45]. It has taken more than 40 years of time and great deal of painstaking research from the initial discovery of these cytokines by American Orthopaedic Surgeon Marshall Urist [14,26,329] to their approval for clinical use by FDA. A lot of work still needs to be done if more BMPs based tissue engineering constructs are to become available for routine clinical use. It would require elucidation of optimal therapeutic dosage, development of more efficient carriers and better understanding of local bone repair environment [14]. Further research will also be required to better define the variables such as route of administration and ideal scaffold material.

The use of BMPs based delivery systems is still in its early days, but recent clinical studies in humans suggest that a promising future will unravel in development of BMPs based products for orthopaedics, periodontics, maxillofacial surgery and other clinical situations. Up until this point most studies and clinical trials have focused on rhBMP-2 and rhBMP-7 but given that bone regeneration involves intricate interplay of network of molecules, it is likely that use of cocktail of molecules comprising of different BMPs may be more suitable approach than a single molecule [13,28,29,330] and hence there is a great need for research to evaluate this potential approach. Similarly advances in the field of biomaterials will also increase the potential approaches for delivery of BMPs for treatment of bone defects. Novel strategies such as nanoparticles and injectable systems will allow restricted and site specific delivery of BMPs. Systems which could potentially deliver BMPs with angiogenic factors and cells could potentially enhance rate, volume and quality of newly formed bone [13,25]. 

Although there have been limited clinical trials in comparison to a large pool of preclinical studies for evaluation of BMPs for routine clinical application in humans but their results have demonstrated that BMPs are effective and there is evidence to suggest that in some situations their efficacy is comparable to or even better than autografts [45]. Although controversy exists regarding their use due to side effects observed in spinal applications [327]. BMP-7 has been compared to autogenous bone graft harvested from iliac crest, currently considered the gold standard in bone grafting materials. Preclinical, clinical and long-term follow up studies have demonstrated safety and effectiveness of BMP-7 [25,331]. Furthermore, prospective clinical trials evaluating the effectiveness of rhBMP-2 and rhBMP-7 for treatment of bone fractures and non-unions have also shown very encouraging results. It is only pertinent that more research is done to expound the relative effectiveness of BMPs, interaction among different types of BMPs and characteristics of the cells responding to BMP signaling. Additionally, there is a great need to distinguish if there is a single pathway to efficient bone regeneration or different clinical scenarios that require more specific tissue engineering approaches. This would enable us to better understand the physiological process involved in bone healing allowing us to develop more efficient and effective tissue-engineered bone constructs.

The principles of inter-species BMP dose extrapolation are not completely understood currently and are applied with varying success in clinical scenarios. Simple scaling of drug doses used in preclinical experimental animal models to humans can be erroneous and misleading. The physicochemical properties of the BMPs used and/or the knowledge of interspecies differences in physiology can be used to improve drug dosing. However, differences in BMP transport via carrier scaffolds, the dose–response relationship and metabolism makes the assessment of accurate BMP dosing for clinical applications very difficult. The reported adverse effects of BMP clinical use give rise to several imperative questions that remain to be addressed. One challenge is that BMP therapeutics use microgram amounts while endogenous BMPs act within the nanogram level. Development of smarter biomaterial carrier for delivery of BMPs and other growth factors in a better-controlled fashion is required. The ever increasing applications for use of BMPs reaffirms that future of regenerative medicine, particularly of BMPs for bone tissue engineering is a bright one [13,18] and possibility of a tissue engineered bone construct as an alternate to autogenous bone graft may be a reality in not so distant future.

## References

[1] Damien C.J., Parsons J.R. (1991). Bone graft and bone graft substitutes: A review of current technology and applications. J. Appl. Biomater..

[2] Javaid M.A., Kaartinen M.T. (2013). Mesenchymal stem cell-based bone tissue engineering. Int. Dent. J. Stud. Res..

[3] Hubble M.J.W. (2002). Bone grafts. Surg. Technol. Int..

[4] Sheikh Z.A.A., Javaid M.A., Abdallah M.N., Khurshid Z.S.Z. (2013). Bone replacement graft materials in dentistry. Dental Biomaterials (Principle and Its Application).

[5] Cypher T.J., Grossman J.P. (1996). Biological principles of bone graft healing. J. Foot Ankle Surg..

[6] Heary R.F., Schlenk R.P., Sacchieri T.A., Barone D., Brotea C. (2002). Persistent iliac crest donor site pain: Independent outcome assessment. Neurosurgery.

[7] Ahlmann E., Patzakis M., Roidis N., Shepherd L., Holtom P. (2002). Comparison of anterior and posterior iliac crest bone grafts in terms of harvest-site morbidity and functional outcomes. J. Bone Jt. Surg. Am..

[8] Robertson P.A., Wray A.C. (2001). Natural history of posterior iliac crest bone graft donation for spinal surgery: A prospective analysis of morbidity. Spine.

[9] Boden S.D. (2005). The ABCs of BMPs. Orthop. Nurs..

[10] Tevlin R., McArdle A., Atashroo D., Walmsley G.G., Senarath-Yapa K., Zielins E.R., Paik K.J., Longaker M.T., Wan D.C. (2014). Biomaterials for craniofacial bone engineering. J. Dent. Res..

[11] LeGeros R.Z. (1997). LeGeros. Bone Substitute Materials and Their Properties.

[12] Arrington E.D., Smith W.J., Chambers H.G., Bucknell A.L., Davino N.A. (1996). Complications of iliac crest bone graft harvesting. Clin. Orthop. Relat. Res..

[13] Bessa P.C., Casal M., Reis R.L. (2008). Bone morphogenetic proteins in tissue engineering: The road from laboratory to clinic, part II (BMP delivery). J. Tissue Eng. Regen. Med..

[14] Termaat M., Den Boer F., Bakker F., Patka P., Haarman H.T.M. (2005). Bone morphogenetic proteins development and clinical efficacy in the treatment of fractures and bone defects. J. Bone Jt. Surg..

[15] Schmitt J.M., Hwang K., Winn S.R., Hollinger J.O. (1999). Bone morphogenetic proteins: An update on basic biology and clinical relevance. J. Orthop. Res..

[16] Reddi A.H. (1998). Role of morphogenetic proteins in skeletal tissue engineering and regeneration. Nat. Biotechnol..

[17] Bragdon B., Moseychuk O., Saldanha S., King D., Julian J., Nohe A. (2011). Bone morphogenetic proteins: A critical review. Cell. Signal..

[18] Carreira A.C., Lojudice F.H., Halcsik E., Navarro R.D., Sogayar M.C., Granjeiro J.M. (2014). Bone morphogenetic proteins: Facts, challenges, and future perspectives. J. Dent. Res..

[19] Urist M.R., Strates B.S. (1971). Bone morphogenetic protein. J. Dent. Res..

[20] Laurencin C.T., Lo K.W. (2013). Bone morphogenetic proteins for bone regeneration and their alternatives. Curr. Pharm. Des..

[21] Docherty Skogh A.C., Engstrand T. (2009). Bone morphogenetic proteins in cranial reconstructions: Clinical evaluation of heparin-chitosan as a carrier for BMP-2. Plast. Reconstr. Surg..

[22] Chenard K.E., Teven C.M., He T.C., Reid R.R. (2012). Bone morphogenetic proteins in craniofacial surgery: Current techniques, clinical experiences, and the future of personalized stem cell therapy. J. Biomed. Biotechnol..

[23] Argintar E., Edwards S., Delahay J. (2011). Bone morphogenetic proteins in orthopaedic trauma surgery. Injury.

[24] Rao S.M., Ugale G.M., Warad S.B. (2013). Bone morphogenetic proteins: Periodontal regeneration. N. Am. J. Med. Sci..

[25] Carreira A.C., Alves G.G., Zambuzzi W.F., Sogayar M.C., Granjeiro J.M. (2014). Bone morphogenetic proteins: Structure, biological function and therapeutic applications. Arch. Biochem. Biophys..

[26] Granjeiro J.M., Oliveira R.C., Bustos-Valenzuela J.C., Sogayar M.C., Taga R. (2005). Bone morphogenetic proteins: From structure to clinical use. Braz. J. Med. Biol. Res..

[27] Myllyla R.M., Haapasaari K.M., Lehenkari P., Tuukkanen J. (2014). Bone morphogenetic proteins 4 and 2/7 induce osteogenic differentiation of mouse skin derived fibroblast and dermal papilla cells. Cell Tissue Res..

[28] Zhang X., Guo J., Zhou Y., Wu G. (2014). The roles of bone morphogenetic proteins and their signaling in the osteogenesis of adipose-derived stem cells. Tissue Eng. B Rev..

[29] Rengachary S.S. (2002). Bone morphogenetic proteins: Basic concepts. Neurosurg. Focus.

[30] Ducy P., Karsenty G. (2000). The family of bone morphogenetic proteins. Kidney Int..

[31] Constam D.B., Robertson E.J. (1999). Regulation of bone morphogenetic protein activity by pro domains and proprotein convertases. J. Cell Biol..

[32] Wordinger R.J., Agarwal R., Talati M., Fuller J., Lambert W., Clark A.F. (2002). Expression of bone morphogenetic proteins (BMP), BMP receptors, and BMP associated proteins in human trabecular meshwork and optic nerve head cells and tissues. Mol. Vis..

[33] Yonemori K., Imamura T., Ishidou Y., Okano T., Matsunaga S., Yoshida H., Kato M., Sampath T.K., Miyazono K., Ten Dijke P. (1997). Bone morphogenetic protein receptors and activin receptors are highly expressed in ossified ligament tissues of patients with ossification of the posterior longitudinal ligament. Am. J. Pathol..

[34] Heldin C.H., Miyazono K., ten Dijke P. (1997). TGF-beta signalling from cell membrane to nucleus through SMAD proteins. Nature.

[35] Huse K., Bakkebø M., Wälchli S., Oksvold M.P., Hilden V.I., Forfang L., Bredahl M.L., Liestøl K., Alizadeh A.A., Smeland E.B. (2012). Role of smad proteins in resistance to BMP-induced growth inhibition in B-cell lymphoma. PLoS One.

[36] Gautschi O.P., Frey S.P., Zellweger R. (2007). Bone morphogenetic proteins in clinical applications. ANZ J. Surg..

[37] Yamaguchi A., Katagiri T., Ikeda T., Wozney J.M., Rosen V., Wang E.A., Kahn A.J., Suda T., Yoshiki S. (1991). Recombinant human bone morphogenetic protein-2 stimulates osteoblastic maturation and inhibits myogenic differentiation *in vitro*. J. Cell Biol..

[38] Thies R.S., Bauduy M., Ashton B.A., Kurtzberg L., Wozney J.M., Rosen V. (1992). Recombinant human bone morphogenetic protein-2 induces osteoblastic differentiation in w-20-17 stromal cells. Endocrinology.

[39] Chen T.L., Bates R.L., Dudley A., Hammonds R.G., Amento E.P. (1991). Bone morphogenetic protein-2b stimulation of growth and osteogenic phenotypes in rat osteoblast-like cells: Comparison with TGF-beta 1. J. Bone Miner. Res..

[40] Mayer H., Scutt A.M., Ankenbauer T. (1996). Subtle differences in the mitogenic effects of recombinant human bone morphogenetic proteins -2 to -7 on DNA synthesis on primary bone-forming cells and identification of BMP-2/4 receptor. Calcif. Tissue Int..

[41] Hughes F.J., Collyer J., Stanfield M., Goodman S.A. (1995). The effects of bone morphogenetic protein-2, -4, and -6 on differentiation of rat osteoblast cells *in vitro*. Endocrinology.

[42] Cheng H., Jiang W., Phillips F.M., Haydon R.C., Peng Y., Zhou L., Luu H.H., An N., Breyer B., Vanichakarn P. (2003). Osteogenic activity of the fourteen types of human bone morphogenetic proteins (BMPS). J. Bone Jt. Surg. Am..

[43] Kang Q., Sun M.H., Cheng H., Peng Y., Montag A.G., Deyrup A.T., Jiang W., Luu H.H., Luo J., Szatkowski J.P. (2004). Characterization of the distinct orthotopic bone-forming activity of 14 BMPs using recombinant adenovirus-mediated gene delivery. Gene Ther..

[44] Katanec D., Granic M., Majstorovic M., Trampus Z., Panduric D.G. (2014). Use of recombinant human bone morphogenetic protein (rhBMP2) in bilateral alveolar ridge augmentation: Case report. Coll. Antropol..

[45] Bibbo C., Nelson J., Ehrlich D., Rougeux B. (2015). Bone morphogenetic proteins: Indications and uses. Clin. Podiatr. Med. Surg..

[46] Mont M.A., Ragland P.S., Biggins B., Friedlaender G., Patel T., Cook S., Etienne G., Shimmin A., Kildey R., Rueger D.C. (2004). Use of bone morphogenetic proteins for musculoskeletal applicationsan overview. J. Bone Jt. Surg..

[47] Cha J.K., Lee J.S., Kim M.S., Choi S.H., Cho K.S., Jung U.W. (2014). Sinus augmentation using BMP-2 in a bovine hydroxyapatite/collagen carrier in dogs. J. Clin. Periodontol..

[48] Martinez-Sanz E., Alkhraisat M.H., Paradas I., Lopez Y., Maldonado E., Gonzalez-Meli B., Berenguer B., Lopez-Cabarcos E., Martinez M.L., Martinez-Alvarez C. (2012). Osteoinduction in the palatal submucosa by injecting BMP-2 on 2 different carriers. J. Craniofac. Surg..

[49] Kempen D.H., Yaszemski M.J., Heijink A., Hefferan T.E., Creemers L.B., Britson J., Maran A., Classic K.L., Dhert W.J., Lu L. (2009). Non-invasive monitoring of BMP-2 retention and bone formation in composites for bone tissue engineering using SPECT/CT and scintillation probes. J. Control. Release.

[50] Gruber R.M., Krohn S., Mauth C., Dard M., Molenberg A., Lange K., Perske C., Schliephake H. (2014). Mandibular reconstruction using a calcium phosphate/polyethylene glycol hydrogel carrier with BMP-2. J. Clin. Periodontol..

[51] Osidak E.O., Osidak M.S., Sivogrivov D.E., Portnaya T.S., Grunina T.M., Galushkina Z.M., Lunin V.G., Karyagina A.S., Domogatskii S.P. (2014). Kinetics of BMP-2 release from collagen carrier: Evaluation by enzyme immunoassay in the presence of plasma proteins. Bull. Exp. Biol. Med..

[52] Ansari S., Freire M.O., Pang E.K., Abdelhamid A.I., Almohaimeed M., Zadeh H.H. (2014). Immobilization of murine anti-BMP-2 monoclonal antibody on various biomaterials for bone tissue engineering. BioMed. Res. Int..

[53] Xie G., Sun J., Zhong G., Liu C., Wei J. (2010). Hydroxyapatite nanoparticles as a controlled-release carrier of BMP-2: Absorption and release kinetics *in vitro*. J. Mater. Sci. Mater. Med..

[54] Horasawa N., Yamashita T., Uehara S., Udagawa N. (2015). High-performance scaffolds on titanium surfaces: Osteoblast differentiation and mineralization promoted by a globular fibrinogen layer through cell-autonomous BMP signaling. Mater. Sci. Eng. C Mater. Biol. Appl..

[55] Hulsart-Billstrom G., Hu Q., Bergman K., Jonsson K.B., Aberg J., Tang R., Larsson S., Hilborn J. (2011). Calcium phosphates compounds in conjunction with hydrogel as carrier for BMP-2: A study on ectopic bone formation in rats. Acta Biomater..

[56] Jun S.H., Lee E.J., Jang T.S., Kim H.E., Jang J.H., Koh Y.H. (2013). Bone morphogenic protein-2 (BMP-2) loaded hybrid coating on porous hydroxyapatite scaffolds for bone tissue engineering. J. Mater. Sci. Mater. Med..

[57] Ji Y., Xu G.P., Zhang Z.P., Xia J.J., Yan J.L., Pan S.H. (2010). BMP-2/PLGA delayed-release microspheres composite graft, selection of bone particulate diameters, and prevention of aseptic inflammation for bone tissue engineering. Ann. Biomed. Eng..

[58] Kempen D.H., Lu L., Hefferan T.E., Creemers L.B., Maran A., Classic K.L., Dhert W.J., Yaszemski M.J. (2008). Retention of *in vitro* and *in vivo* BMP-2 bioactivities in sustained delivery vehicles for bone tissue engineering. Biomaterials.

[59] Murakami N., Saito N., Takahashi J., Ota H., Horiuchi H., Nawata M., Okada T., Nozaki K., Takaoka K. (2003). Repair of a proximal femoral bone defect in dogs using a porous surfaced prosthesis in combination with recombinant BMP-2 and a synthetic polymer carrier. Biomaterials.

[60] Sohier J., Daculsi G., Sourice S., de Groot K., Layrolle P. (2010). Porous beta tricalcium phosphate scaffolds used as a BMP-2 delivery system for bone tissue engineering. J. Biomed. Mater. Res. A.

[61] Kessler E., Takahara K., Biniaminov L., Brusel M., Greenspan D.S. (1996). Bone morphogenetic protein-1: the type I procollagen C-proteinase. Science.

[62] Zhang H., Bradley A. (1996). Mice deficient for BMP2 are nonviable and have defects in amnion/chorion and cardiac development. Development.

[63] Bostrom M.P., Lane J.M., Berberian W.S., Missri A.A., Tomin E., Weiland A., Doty S.B., Glaser D., Rosen V.M. (1995). Immunolocalization and expression of bone morphogenetic proteins 2 and 4 in fracture healing. J. Orthop. Res..

[64] Bahamonde M.E., Lyons K.M. (2001). BMP3: To be or not to be a BMP. J. Bone Jt. Surg. Ser. A.

[65] Cunningham N.S., Jenkins N.A., Gilbert D.J., Copeland N.G., Reddi A.H., Lee S.J. (1995). Growth/differentiation factor-10: A new member of the transforming growth factor-β superfamily related to bone morphogenetic protein-3. Growth Factors.

[66] Ten Dijke P., Yamashita H., Sampath T.K., Reddi A.H., Estevez M., Riddle D.L., Ichijo H., Heldin C.H., Miyazono K. (1994). Identification of type I receptors for osteogenic protein-1 and bone morphogenetic protein-4. J. Biol. Chem..

[67] Xu P.X., Zheng W., Huang L., Maire P., Laclef C., Silvius D. (2003). Six1 is required for the early organogenesis of mammalian kidney. Development.

[68] Winnier G., Blessing M., Labosky P.A., Hogan B.L.M. (1995). Bone morphogenetic protein-4 is required for mesoderm formation and patterning in the mouse. Genes Dev..

[69] Ro T.B., Holt R.U., Brenne A.T., Hjorth-Hansen H., Waage A., Hjertner O., Sundan A., Borset M. (2004). Bone morphogenetic protein-5, -6 and -7 inhibit growth and induce apoptosis in human myeloma cells. Oncogene.

[70] King J.A., Marker P.C., Seung K.J., Kingsley D.M. (1994). BMP5 and the molecular, skeletal, and soft-tissue alterations in short ear mice. Dev. Biol..

[71] Nonner D., Barrett E.F., Kaplan P., Barrett J.N. (2001). Bone morphogenetic proteins (BMP6 and BMP7) enhance the protective effect of neurotrophins on cultured septal cholinergic neurons during hypoglycemia. J. Neurochem..

[72] Hahn G.V., Cohen R.B., Wozney J.M., Levitz C.L., Shore E.M., Zasloff M.A., Kaplan F.S. (1992). A bone morphogenetic protein subfamily: Chromosomal localization of human genes for BMP5, BMP6, and BMP7. Genomics.

[73] Zhang Y., Ge G., Greenspan D.S. (2006). Inhibition of bone morphogenetic protein 1 by native and altered forms of α_2_-macroglobulin. J. Biol. Chem..

[74] Dudley A.T., Lyons K.M., Robertson E.J. (1995). A requirement for bone morphogenetic protein-7 during development of the mammalian kidney and eye. Genes Dev..

[75] Nakase T., Yoshikawa H. (2006). Potential roles of bone morphogenetic proteins (BMPs) in skeletal repair and regeneration. J. Bone Miner. Metab..

[76] Zhao G.Q., Hogan B.L.M. (1996). Evidence that mouse BMP8α (op2) and BMP8b are duplicated genes that play a role in spermatogenesis and placental development. Mech. Dev..

[77] David L., Mallet C., Mazerbourg S., Feige J.J., Bailly S. (2007). Identification of BMP9 and BMP10 as functional activators of the orphan activin receptor-like kinase 1 (ALK1) in endothelial cells. Blood.

[78] Nakashima M., Tachibana K., Iohara K., Ito M., Ishikawa M., Akamine A. (2003). Induction of reparative dentin formation by ultrasound-mediated gene delivery of growth/differentiation factor 11. Hum. Gene Ther..

[79] Furuya K., Nifuji A., Rosen V., Noda M. (1999). Effects of GDF7/BMP12 on proliferation and alkaline phosphatase expression in rat osteoblastic osteosarcoma ROS 17/2.8 cells. J. Cell. Biochem..

[80] Nochi H., Jin H.S., Lou J., Adkisson H.D., Maloney W.J., Hruska K.A. (2004). Adenovirus mediated BMP-13 gene transfer induces chondrogenic differentiation of murine mesenchymal progenitor cells. J. Bone Miner. Res..

[81] Wolfman N.M., Hattersley G., Cox K., Celeste A.J., Nelson R., Yamaji N., Dube J.L., DiBlasio-Smith E., Nove J., Song J.J. (1997). Ectopic induction of tendon and ligament in rats by growth and differentiation factors 5, 6, and 7, members of the TGF-β gene family. J. Clin. Investig..

[82] Di Pasquale E., Rossetti R., Marozzi A., Bodega B., Borgato S., Cavallo L., Einaudi S., Radetti G., Russo G., Sacco M. (2006). Identification of new variants of human BMP15 gene in a large cohort of women with premature ovarian failure. J. Clin. Endocrinol. Metab..

[83] Feiner N., Begemann G., Renz A.J., Meyer A., Kuraku S. (2009). The origin of BMP16, a novel BMP2/4 relative, retained in teleost fish genomes. BMC Evolut. Biol..

[84] Celeste A.J., Murray B.L. (2002). Bone Morphogenetic Protein (BMP)-17 and BMP-18 Compositions. U.S. Patent.

[85] Chen D., Zhao M., Mundy G.R. (2004). Bone morphogenetic proteins. Growth Factors.

[86] Bessa P.C., Casal M., Reis R.L. (2008). Bone morphogenetic proteins in tissue engineering: The road from the laboratory to the clinic, part I (basic concepts). J. Tissue Eng. Regen. Med..

[87] Groeneveld E., Burger E. (2000). Bone morphogenetic proteins in human bone regeneration. Eur. J. Endocrinol..

[88] Issa J.P., Bentley M.V., Iyomasa M.M., Sebald W., de Albuquerque R.F. (2008). Sustained release carriers used to delivery bone morphogenetic proteins in the bone healing process. Anat. Histol. Embryol..

[89] David L., Feige J.J., Bailly S. (2009). Emerging role of bone morphogenetic proteins in angiogenesis. Cytokine Growth Factor Rev..

[90] Degat M.C., Ferreira E., Logeart-Avramoglou D. (2005). Use of growth factors in the repair of bone. Pathologie-Biologie.

[91] Wikesjo U.M., Xiropaidis A.V., Thomson R.C., Cook A.D., Selvig K.A., Hardwick W.R. (2003). Periodontal repair in dogs: rhBMP-2 significantly enhances bone formation under provisions for guided tissue regeneration. J. Clin. Periodontol..

[92] Lippman C.R., Hajjar M., Abshire B., Martin G., Engelman R.W., Cahill D.W. (2004). Cervical spine fusion with bioabsorbable cages. Neurosurg. Focus.

[93] Blunk T., Sieminski A.L., Appel B., Croft C., Courter D.L., Chieh J.J., Goepferich A., Khurana J.S., Gooch K.J. (2003). Bone morphogenetic protein 9: A potent modulator of cartilage development *in vitro*. Growth Factors.

[94] Zheng Y.X., Zhao H.Y., Jing X.B., Qin Q.L., Gu J.J., Tian N., Huang D.P. (2006). Reconstruction of orbital floor defect with polylacticglycolide acid/recombinant human bone morphogenetic protein 2 compound implanted material in sheep. Chin. J. Ophthalmol..

[95] Pan Z.X., Zhang H.X., Wang Y.X., Zhai L.D., Du W. (2014). Effect of recombinant human bone morphogenetic protein 2/poly-lactide-co-glycolic acid (rhBMP-2/PLGA) with core decompression on repair of rabbit femoral head necrosis. Asian Pac. J. Trop. Med..

[96] Qiao C., Zhang K., Jin H., Miao L., Shi C., Liu X., Yuan A., Liu J., Li D., Zheng C. (2013). Using poly(lactic-co-glycolic acid) microspheres to encapsulate plasmid of bone morphogenetic protein 2/polyethylenimine nanoparticles to promote bone formation *in vitro* and *in vivo*. Int. J. Nanomed..

[97] Jones A.A., Buser D., Schenk R., Wozney J., Cochran D.L. (2006). The effect of rhBMP-2 around endosseous implants with and without membranes in the canine model. J. Periodontol..

[98] Shimazu C., Hara T., Kinuta Y., Moriya K., Maruo Y., Hanada S., Minagi S. (2006). Enhanced vertical alveolar bone augmentation by recombinant human bone morphogenetic protein-2 with a carrier in rats. J. Oral Rehabil..

[99] Hu J.J., Jin D., Quan D.P., Zhong S.Z., Chen J.H., Wei K.H., Zhao J., Pei G.X. (2005). Bone defect repair with a new tissue-engineered bone carrying bone morphogenetic protein in rabbits. Acad. J. First Med. Coll. PLA.

[100] Mayer M., Hollinger J., Ron E., Wozney J. (1996). Maxillary alveolar cleft repair in dogs using recombinant human bone morphogenetic protein-2 and a polymer carrier. Plast. Reconstr. Surg..

[101] Kokubo S., Fujimoto R., Yokota S., Fukushima S., Nozaki K., Takahashi K., Miyata K. (2003). Bone regeneration by recombinant human bone morphogenetic protein-2 and a novel biodegradable carrier in a rabbit ulnar defect model. Biomaterials.

[102] Han F., Zhou F., Yang X., Zhao J., Zhao Y., Yuan X. (2014). A pilot study of conically graded chitosan-gelatin hydrogel/PLGA scaffold with dual-delivery of TGF-β1 and BMP-2 for regeneration of cartilage-bone interface. J. Biomed. Mater. Res. Part B Appl. Biomater..

[103] Kawamoto T., Motohashi N., Kitamura A., Baba Y., Suzuki S., Kuroda T. (2003). Experimental tooth movement into bone induced by recombinant human bone morphogenetic protein-2. Cleft Palate-Craniofac. J..

[104] Fan Z., Cao Y., Zhang Z., Zhang M., Lu W., Tang L., Yao Q., Lu G. (2012). Experimental study on application recombinant human bone morphogenetic protein 2(rhBMP-2)/poly-lactide-co-glycolic acid (PLGA)/fibrin sealant(FS) on repair of rabbit radial bone defect. J. Biomed. Eng..

[105] Jeon O., Song S.J., Kang S.W., Putnam A.J., Kim B.S. (2007). Enhancement of ectopic bone formation by bone morphogenetic protein-2 released from a heparin-conjugated poly(l-lactic-co-glycolic acid) scaffold. Biomaterials.

[106] He X.B., Lu W.Z., Tang K.L., Yang L., He J., Zhu Q.H., Liu X.D., Xu J.Z. (2003). Effects of bone morphogenetic protein and transforming growth fractor-beta on biomechanical property for fracture healing in rabbit ulna. Chin. J. Reparative Reconstr. Surg..

[107] Hao W., Dong J., Jiang M., Wu J., Cui F., Zhou D. (2010). Enhanced bone formation in large segmental radial defects by combining adipose-derived stem cells expressing bone morphogenetic protein 2 with nHA/RHLC/PLA scaffold. Int. Orthop..

[108] Chang P.C., Liu B.Y., Liu C.M., Chou H.H., Ho M.H., Liu H.C., Wang D.M., Hou L.T. (2007). Bone tissue engineering with novel rhBMP2-PLLA composite scaffolds. J. Biomed. Mater. Res. A.

[109] Tian W., Bao C., Liu L., Tang W., Zheng X., Li S., Xiong C. (2004). Experimental study on the fabrication of bioactive membrane for inducing bone regeneration. J. Biomed. Eng..

[110] Ji Y., Xu G.P., Yan J.L., Pan S.H. (2009). Transplanted bone morphogenetic protein/poly(lactic-co-glycolic acid) delayed-release microcysts combined with rat micromorselized bone and collagen for bone tissue engineering. J. Int. Med. Res..

[111] Murakami M., Ohtake T., Dorschner R., Gallo R. (2002). Cathelicidin antimicrobial peptides are expressed in salivary glands and saliva. J. Dent. Res..

[112] Suzuki Y., Tanihara M., Suzuki K., Saitou A., Sufan W., Nishimura Y. (2000). Alginate hydrogel linked with synthetic oligopeptide derived from BMP-2 allows ectopic osteoinduction *in vivo*. J. Biomed. Mater. Res..

[113] Kato M., Namikawa T., Terai H., Hoshino M., Miyamoto S., Takaoka K. (2006). Ectopic bone formation in mice associated with a lactic acid/dioxanone/ethylene glycol copolymer-tricalcium phosphate composite with added recombinant human bone morphogenetic protein-2. Biomaterials.

[114] Lutolf M.P., Lauer-Fields J.L., Schmoekel H.G., Metters A.T., Weber F.E., Fields G.B., Hubbell J.A. (2003). Synthetic matrix metalloproteinase-sensitive hydrogels for the conduction of tissue regeneration: Engineering cell-invasion characteristics. Proc. Natl. Acad. Sci. USA.

[115] Lutolf M.P., Weber F.E., Schmoekel H.G., Schense J.C., Kohler T., Muller R., Hubbell J.A. (2003). Repair of bone defects using synthetic mimetics of collagenous extracellular matrices. Nat. Biotechnol..

[116] Rizzi S.C., Ehrbar M., Halstenberg S., Raeber G.P., Schmoekel H.G., Hagenmuller H., Muller R., Weber F.E., Hubbell J.A. (2006). Recombinant protein-co-peg networks as cell-adhesive and proteolytically degradable hydrogel matrixes. Part II: Biofunctional characteristics. Biomacromolecules.

[117] Kinard L.A., Chu C.Y., Tabata Y., Kasper F.K., Mikos A.G. (2013). Bone morphogenetic protein-2 release from composite hydrogels of oligo(poly(ethylene glycol) fumarate) and gelatin. Pharm. Res..

[118] Pratt A.B., Weber F.E., Schmoekel H.G., Muller R., Hubbell J.A. (2004). Synthetic extracellular matrices for *in situ* tissue engineering. Biotechnol. Bioeng..

[119] Gao T., Uludag H. (2001). Effect of molecular weight of thermoreversible polymer on *in vivo* retention of rhBMP-2. J. Biomed. Mater. Res..

[120] Schmoekel H., Schense J.C., Weber F.E., Gratz K.W., Gnagi D., Muller R., Hubbell J.A. (2004). Bone healing in the rat and dog with nonglycosylated BMP-2 demonstrating low solubility in fibrin matrices. J. Orthop. Res..

[121] Schmoekel H.G., Weber F.E., Hurter K., Schense J.C., Seiler G., Ryrz U., Spreng D., Schawalder P., Hubbell J. (2005). Enhancement of bone healing using non-glycosylated rhBMP-2 released from a fibrin matrix in dogs and cats. J. Small Anim. Pract..

[122] Schmoekel H.G., Weber F.E., Schense J.C., Gratz K.W., Schawalder P., Hubbell J.A. (2005). Bone repair with a form of BMP-2 engineered for incorporation into fibrin cell ingrowth matrices. Biotechnol. Bioeng..

[123] Zhu S.J., Choi B.H., Huh J.Y., Jung J.H., Kim B.Y., Lee S.H. (2006). A comparative qualitative histological analysis of tissue-engineered bone using bone marrow mesenchymal stem cells, alveolar bone cells, and periosteal cells. Oral Surg. Oral Med. Oral Pathol. Oral Radiol. Endod..

[124] Zhu S.J., Choi B.H., Jung J.H., Lee S.H., Huh J.Y., You T.M., Lee H.J., Li J. (2006). A comparative histologic analysis of tissue-engineered bone using platelet-rich plasma and platelet-enriched fibrin glue. Oral Surg. Oral Med. Oral Pathol. Oral Radiol. Endod..

[125] Arnander C., Westermark A., Veltheim R., Docherty-Skogh A.C., Hilborn J., Engstrand T. (2006). Three-dimensional technology and bone morphogenetic protein in frontal bone reconstruction. J. Craniofac. Surg..

[126] Cui G., Li J., Lei W. (2007). Effect of injectable fibrin sealant compounded with bone morphogenetic protein on proliferation and differentiation of marrow stromal cells towards osteoblasts in rabbits. Chin. J. Reparative Reconstr. Surg..

[127] Patel V.V., Zhao L., Wong P., Kanim L., Bae H.W., Pradhan B.B., Delamarter R.B. (2006). Controlling bone morphogenetic protein diffusion and bone morphogenetic protein-stimulated bone growth using fibrin glue. Spine.

[128] Yang H.S., La W.G., Cho Y.M., Shin W., Yeo G.D., Kim B.S. (2012). Comparison between heparin-conjugated fibrin and collagen sponge as bone morphogenetic protein-2 carriers for bone regeneration. Exp. Mol. Med..

[129] Hong J.Y., Kang S.W., Kim J.W., Suh S.W., Ko Y.J., Park J.H. (2014). Optimal condition of heparin-conjugated fibrin with bone morphogenetic protein-2 for spinal fusion in a rabbit model. Cytotherapy.

[130] Koo K.H., Lee J.M., Ahn J.M., Kim B.S., La W.G., Kim C.S., Im G.I. (2013). Controlled delivery of low-dose bone morphogenetic protein-2 using heparin-conjugated fibrin in the posterolateral lumbar fusion of rabbits. Artif. Organs.

[131] Hong L., Tabata Y., Yamamoto M., Miyamoto S., Yamada K., Hashimoto N., Ikada Y. (1998). Comparison of bone regeneration in a rabbit skull defect by recombinant human BMP-2 incorporated in biodegradable hydrogel and in solution. J. Biomater. Sci. Polym. Ed..

[132] Takahashi Y., Yamamoto M., Yamada K., Kawakami O., Tabata Y. (2007). Skull bone regeneration in nonhuman primates by controlled release of bone morphogenetic protein-2 from a biodegradable hydrogel. Tissue Eng..

[133] Yamamoto M., Ikada Y., Tabata Y. (2001). Controlled release of growth factors based on biodegradation of gelatin hydrogel. J. Biomater. Sci. Polym. Ed..

[134] Yamamoto M., Takahashi Y., Tabata Y. (2003). Controlled release by biodegradable hydrogels enhances the ectopic bone formation of bone morphogenetic protein. Biomaterials.

[135] Bulpitt P., Aeschlimann D. (1999). New strategy for chemical modification of hyaluronic acid: Preparation of functionalized derivatives and their use in the formation of novel biocompatible hydrogels. J. Biomed. Mater. Res..

[136] Hulsart-Billstrom G., Yuen P.K., Marsell R., Hilborn J., Larsson S., Ossipov D. (2013). Bisphosphonate-linked hyaluronic acid hydrogel sequesters and enzymatically releases active bone morphogenetic protein-2 for induction of osteogenic differentiation. Biomacromolecules.

[137] Arosarena O., Collins W. (2005). Comparison of BMP-2 and -4 for rat mandibular bone regeneration at various doses. Orthod. Craniofac. Res..

[138] Arosarena O.A., Collins W.L. (2005). Bone regeneration in the rat mandible with bone morphogenetic protein-2: A comparison of two carriers. Otolaryngol. Head Neck Surg..

[139] Hunt D.R., Jovanovic S.A., Wikesjo U.M., Wozney J.M., Bernard G.W. (2001). Hyaluronan supports recombinant human bone morphogenetic protein-2 induced bone reconstruction of advanced alveolar ridge defects in dogs. A pilot study. J. Periodontol..

[140] Seeherman H.J., Bouxsein M., Kim H., Li R., Li X.J., Aiolova M., Wozney J.M. (2004). Recombinant human bone morphogenetic protein-2 delivered in an injectable calcium phosphate paste accelerates osteotomy-site healing in a nonhuman primate model. J. Bone Jt. Surg. Am..

[141] Eckardt H., Christensen K.S., Lind M., Hansen E.S., Hall D.W., Hvid I. (2005). Recombinant human bone morphogenetic protein 2 enhances bone healing in an experimental model of fractures at risk of non-union. Injury.

[142] Vogelin E., Jones N.F., Huang J.I., Brekke J.H., Lieberman J.R. (2005). Healing of a critical-sized defect in the rat femur with use of a vascularized periosteal flap, a biodegradable matrix, and bone morphogenetic protein. J. Bone Jt. Surg. Am..

[143] Karageorgiou V., Tomkins M., Fajardo R., Meinel L., Snyder B., Wade K., Chen J., Vunjak-Novakovic G., Kaplan D.L. (2006). Porous silk fibroin 3-D scaffolds for delivery of bone morphogenetic protein-2 *in vitro* and *in vivo*. J. Biomed. Mater. Res. A.

[144] Karageorgiou V., Meinel L., Hofmann S., Malhotra A., Volloch V., Kaplan D. (2004). Bone morphogenetic protein-2 decorated silk fibroin films induce osteogenic differentiation of human bone marrow stromal cells. J. Biomed. Mater. Res. A.

[145] Li C., Vepari C., Jin H.J., Kim H.J., Kaplan D.L. (2006). Electrospun silk-BMP-2 scaffolds for bone tissue engineering. Biomaterials.

[146] Kirker-Head C., Karageorgiou V., Hofmann S., Fajardo R., Betz O., Merkle H.P., Hilbe M., von Rechenberg B., McCool J., Abrahamsen L. (2007). BMP-silk composite matrices heal critically sized femoral defects. Bone.

[147] Simmons C.A., Alsberg E., Hsiong S., Kim W.J., Mooney D.J. (2004). Dual growth factor delivery and controlled scaffold degradation enhance *in vivo* bone formation by transplanted bone marrow stromal cells. Bone.

[148] Saito A., Suzuki Y., Ogata S., Ohtsuki C., Tanihara M. (2005). Accelerated bone repair with the use of a synthetic BMP-2-derived peptide and bone-marrow stromal cells. J. Biomed. Mater. Res. A.

[149] Saito A., Suzuki Y., Ogata S., Ohtsuki C., Tanihara M. (2004). Prolonged ectopic calcification induced by BMP-2-derived synthetic peptide. J. Biomed. Mater. Res. A.

[150] Saito A., Suzuki Y., Kitamura M., Ogata S., Yoshihara Y., Masuda S., Ohtsuki C., Tanihara M. (2006). Repair of 20-mm long rabbit radial bone defects using BMP-derived peptide combined with an alpha-tricalcium phosphate scaffold. J. Biomed. Mater. Res. A.

[151] Lopez-Lacomba J.L., Garcia-Cantalejo J.M., Sanz Casado J.V., Abarrategi A., Correas Magana V., Ramos V. (2006). Use of rhBMP-2 activated chitosan films to improve osseointegration. Biomacromolecules.

[152] Park Y.J., Kim K.H., Lee J.Y., Ku Y., Lee S.J., Min B.M., Chung C.P. (2006). Immobilization of bone morphogenetic protein-2 on a nanofibrous chitosan membrane for enhanced guided bone regeneration. Biotechnol. Appl. Biochem..

[153] Yang X., Han G., Pang X., Fan M. (2012). Chitosan/collagen scaffold containing bone morphogenetic protein-7 DNA supports dental pulp stem cell differentiation *in vitro* and *in vivo*. J. Biomed. Mater. Res. A.

[154] Park D.J., Choi B.H., Zhu S.J., Huh J.Y., Kim B.Y., Lee S.H. (2005). Injectable bone using chitosan-alginate gel/mesenchymal stem cells/BMP-2 composites. J. Cranio-maxillo-fac. Surg..

[155] Liang D., Zuo A., Wang B., Zhang J. (2005). *In vitro* osteogenesis of the compound of chitosan and recombinant human bone morphogenetic protein 2. Chin. J. Reparative Reconstr. Surg..

[156] Maire M., Chaubet F., Mary P., Blanchat C., Meunier A., Logeart-Avramoglou D. (2005). Bovine BMP osteoinductive potential enhanced by functionalized dextran-derived hydrogels. Biomaterials.

[157] Sumner D.R., Turner T.M., Urban R.M., Virdi A.S., Inoue N. (2006). Additive enhancement of implant fixation following combined treatment with rhTGF-β2 and rhBMP-2 in a canine model. J. Bone Jt. Surg. Am..

[158] Becker J., Kirsch A., Schwarz F., Chatzinikolaidou M., Rothamel D., Lekovic V., Laub M., Jennissen H.P. (2006). Bone apposition to titanium implants biocoated with recombinant human bone morphogenetic protein-2 (rhBMP-2). A pilot study in dogs. Clin. Oral Investig..

[159] Stenport V.F., Johansson C., Heo S.J., Aspenberg P., Albrektsson T. (2003). Titanium implants and BMP-7 in bone: An experimental model in the rabbit. J. Mater. Sci. Mater. Med..

[160] Sun S.X., Guo H.H., Zhang J., Yu B., Sun K.N., Jin Q.H. (2014). BMP-2 and titanium particles synergistically activate osteoclast formation. Braz. J. Med. Biol. Res..

[161] Jensen O.T., Lehman H., Ringeman J.L., Casap N. (2014). Fabrication of printed titanium shells for containment of BMP-2 composite graft materials for alveolar bone reconstruction. Int. J. Oral Maxillofac. Implant..

[162] Sachse A., Wagner A., Keller M., Wagner O., Wetzel W.D., Layher F., Venbrocks R.A., Hortschansky P., Pietraszczyk M., Wiederanders B. (2005). Osteointegration of hydroxyapatite-titanium implants coated with nonglycosylated recombinant human bone morphogenetic protein-2 (BMP-2) in aged sheep. Bone.

[163] Bae S.E., Choi J., Joung Y.K., Park K., Han D.K. (2012). Controlled release of bone morphogenetic protein (BMP)-2 from nanocomplex incorporated on hydroxyapatite-formed titanium surface. J. Control. Release.

[164] Yang D.H., Lee D., Kwon Y., Kim H.J., Chun H.J., Jang J.W., Khang G. (2014). Surface modification of titanium with hydroxyapatite-heparin-BMP-2 enhances the efficacy of bone formation and osseointegration *in vitro* and *in vivo*. J. Tissue Eng. Regen. Med..

[165] Poth N., Seiffart V., Gross G., Menzel H., Dempwolf W. (2015). Biodegradable chitosan nanoparticle coatings on titanium for the delivery of BMP-2. Biomolecules.

[166] Phillips F.M., Turner A.S., Seim H.B., MacLeay J., Toth C.A., Pierce A.R., Wheeler D.L. (2006). *In vivo* BMP-7 (OP-1) enhancement of osteoporotic vertebral bodies in an ovine model. Spine J..

[167] Schrier J.A., Fink B.F., Rodgers J.B., Vasconez H.C., DeLuca P.P. (2001). Effect of a freeze-dried CMC/PLGA microsphere matrix of rhBMP-2 on bone healing. AAPS PharmSciTech.

[168] Oldham J.B., Lu L., Zhu X., Porter B.D., Hefferan T.E., Larson D.R., Currier B.L., Mikos A.G., Yaszemski M.J. (2000). Biological activity of rhBMP-2 released from PLGA microspheres. J. Biomech. Eng..

[169] Lee S.Y., Yun Y.P., Song H.R., Chun H.J., Yang D.H., Park K., Kim S.E. (2013). The effect of titanium with heparin/BMP-2 complex for improving osteoblast activity. Carbohydr. Polym..

[170] Kenley R., Marden L., Turek T., Jin L., Ron E., Hollinger J.O. (1994). Osseous regeneration in the rat calvarium using novel delivery systems for recombinant human bone morphogenetic protein-2 (rhBMP-2). J. Biomed. Mater. Res..

[171] Ruhe P.Q., Boerman O.C., Russel F.G., Spauwen P.H., Mikos A.G., Jansen J.A. (2005). Controlled release of rhBMP-2 loaded poly(dl-lactic-co-glycolic acid)/calcium phosphate cement composites *in vivo*. J. control. Release.

[172] Ruhe P.Q., Boerman O.C., Russel F.G., Mikos A.G., Spauwen P.H., Jansen J.A. (2006). *In vivo* release of rhBMP-2 loaded porous calcium phosphate cement pretreated with albumin. J. Mater. Sci. Mater. Med..

[173] Saitoh H., Takata T., Nikai H., Shintani H., Hyon S.H., Ikada Y. (1994). Effect of polylactic acid on osteoinduction of demineralized bone: Preliminary study of the usefulness of polylactic acid as a carrier of bone morphogenetic protein. J. Oral Rehabil..

[174] Xu X., Yang J., Ding L., Li J. (2015). Bone morphogenetic protein-2-encapsulated grafted-poly-lactic acid-polycaprolactone nanoparticles promote bone repair. Cell Biochem. Biophys..

[175] Wang Y.J., Lin F.H., Sun J.S., Huang Y.C., Chueh S.C., Hsu F.Y. (2003). Collagen-hydroxyapatite microspheres as carriers for bone morphogenic protein-4. Artif. Organs.

[176] Murphy C.M., Schindeler A., Gleeson J.P., Yu N.Y., Cantrill L.C., Mikulec K., Peacock L., O’Brien F.J., Little D.G. (2014). A collagen-hydroxyapatite scaffold allows for binding and co-delivery of recombinant bone morphogenetic proteins and bisphosphonates. Acta Biomater..

[177] Hannink G., Geutjes P.J., Daamen W.F., Buma P. (2013). Evaluation of collagen/heparin coated tcp/ha granules for long-term delivery of BMP-2. J. Mater. Sci. Mater. Med..

[178] Itoh S., Matubara M., Kawauchi T., Nakamura H., Yukitake S., Ichinose S., Shinomiya K. (2001). Enhancement of bone ingrowth in a titanium fiber mesh implant by rhBMP-2 and hyaluronic acid. J. Mater. Sci. Mater. Med..

[179] Chen W., Liu Z., Yue Y., Wan L., Hu J., Lu B. (2012). Preparation of basic fibroblast growth factor chitosan microsphere and its properties. Chin. J. Reparative Reconstr. Surg..

[180] Chen F.M., Wu Z.F., Sun H.H., Wu H., Xin S.N., Wang Q.T., Dong G.Y., Ma Z.W., Huang S., Zhang Y.J. (2006). Release of bioactive BMP from dextran-derived microspheres: A novel delivery concept. Int. J. Pharm..

[181] Chen F.M., Zhao Y.M., Zhang R., Jin T., Sun H.H., Wu Z.F., Jin Y. (2007). Periodontal regeneration using novel glycidyl methacrylated dextran (Dex-GMA)/gelatin scaffolds containing microspheres loaded with bone morphogenetic proteins. J. Control. Release.

[182] Qin Y., Pei G.X., Xie D.M., Jin D., Wei K.H. (2003). Effect of bone morphogenetic protein microspheres on biological behavior of rabbit bone marrow stem cells. Acad. J. First Med. Coll. PLA.

[183] Aebli N., Stich H., Schawalder P., Theis J.C., Krebs J. (2005). Effects of bone morphogenetic protein-2 and hyaluronic acid on the osseointegration of hydroxyapatite-coated implants: An experimental study in sheep. J. Biomed. Mater. Res. A.

[184] Zhang C., Hu Y., Xiong Z., Zhang S., Yan Y., Cui F. (2005). Tissue engineered bone regeneration of periosteal cells using recombinant human bone morphogenetic protein 2 induce. Chin. J. Reparative Reconstr. Surg..

[185] Hu Y.Y., Zhang C., Lu R., Xu J.Q., Li D. (2003). Repair of radius defect with bone-morphogenetic-protein loaded hydroxyapatite/collagen-poly(L-lactic acid) composite. Chin. J. Traumatol..

[186] Kaito T., Myoui A., Takaoka K., Saito N., Nishikawa M., Tamai N., Ohgushi H., Yoshikawa H. (2005). Potentiation of the activity of bone morphogenetic protein-2 in bone regeneration by a PLA-PEG/hydroxyapatite composite. Biomaterials.

[187] Yoneda M., Terai H., Imai Y., Okada T., Nozaki K., Inoue H., Miyamoto S., Takaoka K. (2005). Repair of an intercalated long bone defect with a synthetic biodegradable bone-inducing implant. Biomaterials.

[188] Matsushita N., Terai H., Okada T., Nozaki K., Inoue H., Miyamoto S., Takaoka K. (2004). A new bone-inducing biodegradable porous beta-tricalcium phosphate. J. Biomed. Mater. Res. A.

[189] Namikawa T., Terai H., Suzuki E., Hoshino M., Toyoda H., Nakamura H., Miyamoto S., Takahashi N., Ninomiya T., Takaoka K. (2005). Experimental spinal fusion with recombinant human bone morphogenetic protein-2 delivered by a synthetic polymer and beta-tricalcium phosphate in a rabbit model. Spine.

[190] Hong S.J., Kim C.S., Han D.K., Cho I.H., Jung U.W., Choi S.H., Kim C.K., Cho K.S. (2006). The effect of a fibrin-fibronectin/beta-tricalcium phosphate/recombinant human bone morphogenetic protein-2 system on bone formation in rat calvarial defects. Biomaterials.

[191] Sun X.J., Xia L.G., Chou L.L., Zhong W., Zhang X.L., Wang S.Y., Zhao J., Jiang X.Q., Zhang Z.Y. (2010). Maxillary sinus floor elevation using a tissue engineered bone complex with BMP-2 gene modified bmscs and a novel porous ceramic scaffold in rabbits. Arch. Oral Biol..

[192] Abarrategi A., Moreno-Vicente C., Martinez-Vazquez F.J., Civantos A., Ramos V., Sanz-Casado J.V., Martinez-Corria R., Perera F.H., Mulero F., Miranda P. (2012). Biological properties of solid free form designed ceramic scaffolds with BMP-2: *In vitro* and *in vivo* evaluation. PLoS One.

[193] Roldan J.C., Detsch R., Schaefer S., Chang E., Kelantan M., Waiss W., Reichert T.E., Gurtner G.C., Deisinger U. (2010). Bone formation and degradation of a highly porous biphasic calcium phosphate ceramic in presence of BMP-7, VEGF and mesenchymal stem cells in an ectopic mouse model. J. Cranio-Maxillo-Fac. Surg..

[194] Schopper C., Moser D., Spassova E., Goriwoda W., Lagogiannis G., Hoering B., Ewers R., Redl H. (2008). Bone regeneration using a naturally grown ha/tcp carrier loaded with rh BMP-2 is independent of barrier-membrane effects. J. Biomed. Mater. Res. A.

[195] Crouzier T., Sailhan F., Becquart P., Guillot R., Logeart-Avramoglou D., Picart C. (2011). The performance of BMP-2 loaded TCP/HAP porous ceramics with a polyelectrolyte multilayer film coating. Biomaterials.

[196] Tamimi F., Sheikh Z., Barralet J. (2012). Dicalcium phosphate cements: Brushite and monetite. Acta Biomater..

[197] Woodard J.R., Hilldore A.J., Lan S.K., Park C.J., Morgan A.W., Eurell J.A., Clark S.G., Wheeler M.B., Jamison R.D., Wagoner Johnson A.J. (2007). The mechanical properties and osteoconductivity of hydroxyapatite bone scaffolds with multi-scale porosity. Biomaterials.

[198] LeGeros R.Z. (2002). Properties of osteoconductive biomaterials: Calcium phosphates. Clin. Orthop. Relat. Res..

[199] Tsuruga E., Takita H., Itoh H., Wakisaka Y., Kuboki Y. (1997). Pore size of porous hydroxyapatite as the cell-substratum controls BMP-induced osteogenesis. J. Biochem..

[200] Noshi T., Yoshikawa T., Dohi Y., Ikeuchi M., Horiuchi K., Ichijima K., Sugimura M., Yonemasu K., Ohgushi H. (2001). Recombinant human bone morphogenetic protein-2 potentiates the *in vivo* osteogenic ability of marrow/hydroxyapatite composites. Artif. Organs.

[201] Kraiwattanapong C., Boden S.D., Louis-Ugbo J., Attallah E., Barnes B., Hutton W.C. (2005). Comparison of healos/bone marrow to infuse(rhBMP-2/ACS) with a collagen-ceramic sponge bulking agent as graft substitutes for lumbar spine fusion. Spine.

[202] Boden S.D., Martin G.J., Morone M.A., Ugbo J.L., Moskovitz P.A. (1999). Posterolateral lumbar intertransverse process spine arthrodesis with recombinant human bone morphogenetic protein 2/hydroxyapatite-tricalcium phosphate after laminectomy in the nonhuman primate. Spine.

[203] Uludag H., D’Augusta D., Palmer R., Timony G., Wozney J. (1999). Characterization of rhBMP-2 pharmacokinetics implanted with biomaterial carriers in the rat ectopic model. J. Biomed. Mater. Res..

[204] Rohanizadeh R., Chung K. (2011). Hydroxyapatite as a carrier for bone morphogenetic protein. J. Oral Implantol..

[205] Xiao W., Fu H., Rahaman M.N., Liu Y., Bal B.S. (2013). Hollow hydroxyapatite microspheres: A novel bioactive and osteoconductive carrier for controlled release of bone morphogenetic protein-2 in bone regeneration. Acta Biomater..

[206] Zambuzzi W.F., Fernandes G.V., Iano F.G., Fernandes Mda S., Granjeiro J.M., Oliveira R.C. (2012). Exploring anorganic bovine bone granules as osteoblast carriers for bone bioengineering: A study in rat critical-size calvarial defects. Braz. Dent. J..

[207] Mitri F., Alves G., Fernandes G., Konig B., Rossi A.J., Granjeiro J. (2012). Cytocompatibility of porous biphasic calcium phosphate granules with human mesenchymal cells by a multiparametric assay. Artif. Organs.

[208] Lomelino Rde O., Castro S., Alves G.G., Santos S.R., Gameiro V.S., Rossi A.M., Granjeiro J.M. (2012). The association of human primary bone cells with biphasic calcium phosphate (βTCP/HA 70:30) granules increases bone repair. J. Mater. Sci. Mater. Med..

[209] Kamitakahara M., Ohtsuki C., Miyazaki T. (2008). Review paper: Behavior of ceramic biomaterials derived from tricalcium phosphate in physiological condition. J. Biomater. Appl..

[210] Hagi T.T., Wu G., Liu Y., Hunziker E.B. (2010). Cell-mediated BMP-2 liberation promotes bone formation in a mechanically unstable implant environment. Bone.

[211] Yuan H., De Bruijn J., Zhang X., van Blitterswijk C., de Groot K. (2001). Use of an osteoinductive biomaterial as a bone morphogenetic protein carrier. J. Mater. Sci. Mater. Med..

[212] Cao X., Liu C., Chen J. (2006). Experimental studies on the porous calcium phosphate cement combined with recombinant human bone morphogenetic protein 2 for bone defects repair. Chin. J. Reparative Reconstr. Surg..

[213] Edwards R.B., Seeherman H.J., Bogdanske J.J., Devitt J., Vanderby R., Markel M.D. (2004). Percutaneous injection of recombinant human bone morphogenetic protein-2 in a calcium phosphate paste accelerates healing of a canine tibial osteotomy. J. Bone Jt. Surg. Am..

[214] Miranda D.A., Blumenthal N.M., Sorensen R.G., Wozney J.M., Wikesjo U.M. (2005). Evaluation of recombinant human bone morphogenetic protein-2 on the repair of alveolar ridge defects in baboons. J. Periodontol..

[215] Barnes B., Boden S.D., Louis-Ugbo J., Tomak P.R., Park J.S., Park M.S., Minamide A. (2005). Lower dose of rhBMP-2 achieves spine fusion when combined with an osteoconductive bulking agent in non-human primates. Spine.

[216] Saito N., Takaoka K. (2003). New synthetic biodegradable polymers as BMP carriers for bone tissue engineering. Biomaterials.

[217] Saito N., Okada T., Toba S., Miyamoto S., Takaoka K. (1999). New synthetic absorbable polymers as BMP carriers: Plastic properties of poly-D,L-lactic acid-polyethylene glycol block copolymers. J. Biomed. Mater. Res..

[218] Saito N., Okada T., Horiuchi H., Ota H., Takahashi J., Murakami N., Nawata M., Kojima S., Nozaki K., Takaoka K. (2003). Local bone formation by injection of recombinant human bone morphogenetic protein-2 contained in polymer carriers. Bone.

[219] Miyamoto S., Takaoka K., Okada T., Yoshikawa H., Hashimoto J., Suzuki S., Ono K. (1992). Evaluation of polylactic acid homopolymers as carriers for bone morphogenetic protein. Clin. Orthop. Relat. Res..

[220] Polimeni G., Koo K.T., Pringle G.A., Agelan A., Safadi F.F., Wikesjo U.M. (2008). Histopathological observations of a polylactic acid-based device intended for guided bone/tissue regeneration. Clin. Implant Dent. Relat. Res..

[221] Gao T.J., Kousinioris N.A., Wozney J.M., Winn S., Uludag H. (2002). Synthetic thermoreversible polymers are compatible with osteoinductive activity of recombinant human bone morphogenetic protein 2. Tissue Eng..

[222] Saito N., Murakami N., Takahashi J., Horiuchi H., Ota H., Kato H., Okada T., Nozaki K., Takaoka K. (2005). Synthetic biodegradable polymers as drug delivery systems for bone morphogenetic proteins. Adv. Drug Deliv. Rev..

[223] Saito N., Okada T., Horiuchi H., Murakami N., Takahashi J., Nawata M., Ota H., Nozaki K., Takaoka K. (2001). A biodegradable polymer as a cytokine delivery system for inducing bone formation. Nat. Biotechnol..

[224] Suzuki A., Terai H., Toyoda H., Namikawa T., Yokota Y., Tsunoda T., Takaoka K. (2006). A biodegradable delivery system for antibiotics and recombinant human bone morphogenetic protein-2: A potential treatment for infected bone defects. J. Orthop. Res..

[225] Matsushita N., Terai H., Okada T., Nozaki K., Inoue H., Miyamoto S., Takaoka K. (2006). Accelerated repair of a bone defect with a synthetic biodegradable bone-inducing implant. J. Orthop. Sci..

[226] Asahina I., Watanabe M., Sakurai N., Mori M., Enomoto S. (1997). Repair of bone defect in primate mandible using a bone morphogenetic protein (BMP)-hydroxyapatite-collagen composite. J. Med. Dent. Sci..

[227] Li J., Li Y., Ma S., Gao Y., Zuo Y., Hu J. (2010). Enhancement of bone formation by BMP-7 transduced mscs on biomimetic nano-hydroxyapatite/polyamide composite scaffolds in repair of mandibular defects. J. Biomed. Mater. Res. A.

[228] Winet H., Hollinger J.O. (1993). Incorporation of polylactide-polyglycolide in a cortical defect: Neoosteogenesis in a bone chamber. J. Biomed. Mater. Res..

[229] Miller R.A., Brady J.M., Cutright D.E. (1977). Degradation rates of oral resorbable implants (polylactates and polyglycolates): Rate modification with changes in PLA/PGA copolymer ratios. J. Biomed. Mater. Res..

[230] Grayson A.C., Voskerician G., Lynn A., Anderson J.M., Cima M.J., Langer R. (2004). Differential degradation rates *in vivo* and *in vitro* of biocompatible poly(lactic acid) and poly(glycolic acid) homo- and co-polymers for a polymeric drug-delivery microchip. J. Biomater. Sci. Polym. Ed..

[231] Peppas N.A. (1997). Hydrogels and drug delivery. Curr. Opin. Colloid Interface Sci..

[232] Hoare T.R., Kohane D.S. (2008). Hydrogels in drug delivery: Progress and challenges. Polymer.

[233] Kamath K.R., Park K. (1993). Biodegradable hydrogels in drug delivery. Adv. Drug Deliv. Rev..

[234] Vermonden T., Censi R., Hennink W.E. (2012). Hydrogels for protein delivery. Chem. Rev..

[235] Tessmar J.K., Göpferich A.M. (2007). Matrices and scaffolds for protein delivery in tissue engineering. Adv. Drug Deliv. Rev..

[236] Fisher J.P., Jo S., Mikos A.G., Reddi A.H. (2004). Thermoreversible hydrogel scaffolds for articular cartilage engineering. J. Biomed. Mater. Res. A.

[237] Mano J.F., Reis R.L. (2007). Osteochondral defects: Present situation and tissue engineering approaches. J. Tissue Eng. Regen. Med..

[238] Mano J.F., Silva G.A., Azevedo H.S., Malafaya P.B., Sousa R.A., Silva S.S., Boesel L.F., Oliveira J.M., Santos T.C., Marques A.P. (2007). Natural origin biodegradable systems in tissue engineering and regenerative medicine: Present status and some moving trends. J. R. Soc. Interface.

[239] Malafaya P.B., Gomes M.E., Salgado A.J., Reis R.L. (2003). Polymer based scaffolds and carriers for bioactive agents from different natural origin materials. Adv. Exp. Med. Biol..

[240] Gomes M.E., Malafaya P.B., Reis R.L. (2004). Methodologies for processing biodegradable and natural origin scaffolds for bone and cartilage tissue-engineering applications. Methods Mol. Biol..

[241] Saito A., Suzuki Y., Ogata S., Ohtsuki C., Tanihara M. (2003). Activation of osteo-progenitor cells by a novel synthetic peptide derived from the bone morphogenetic protein-2 knuckle epitope. Biochim. Biophys. Acta.

[242] Tuzlakoglu K., Alves C.M., Mano J.F., Reis R.L. (2004). Production and characterization of chitosan fibers and 3-D fiber mesh scaffolds for tissue engineering applications. Macromol. Biosci..

[243] Baran E.T., Mano J.F., Reis R.L. (2004). Starch-chitosan hydrogels prepared by reductive alkylation cross-linking. J. Mater. Sci. Mater. Med..

[244] Prabaharan M., Mano J.F. (2005). Chitosan-based particles as controlled drug delivery systems. Drug Deliv..

[245] Hsieh C.Y., Hsieh H.J., Liu H.C., Wang D.M., Hou L.T. (2006). Fabrication and release behavior of a novel freeze-gelled chitosan/gamma-PGA scaffold as a carrier for rhBMP-2. Dent. Mater..

[246] Hattori T. (1990). Experimental investigations of osteogenesis and chondrogenesis by implant of BMP-fibrin glue mixture. Nihon Seikeigeka Gakkai Zasshi.

[247] Han D.K., Kim C.S., Jung U.W., Chai J.K., Choi S.H., Kim C.K., Cho K.S. (2005). Effect of a fibrin-fibronectin sealing system as a carrier for recombinant human bone morphogenetic protein-4 on bone formation in rat calvarial defects. J. Periodontol..

[248] Ren W., Yang L., Dong S. (2000). The effects of the complex of rhBMP2 and fibrin sealant on dental pulp. Chin. J. Stomatol..

[249] Osathanon T., Linnes M.L., Rajachar R.M., Ratner B.D., Somerman M.J., Giachelli C.M. (2008). Microporous nanofibrous fibrin-based scaffolds for bone tissue engineering. Biomaterials.

[250] Hubbell J.A. (2006). Matrix-bound growth factors in tissue repair. Swiss Med. Wkly..

[251] Kim H.D., Valentini R.F. (2002). Retention and activity of BMP-2 in hyaluronic acid-based scaffolds *in vitro*. J. Biomed. Mater. Res..

[252] Wikesjo U.M., Lim W.H., Thomson R.C., Cook A.D., Wozney J.M., Hardwick W.R. (2003). Periodontal repair in dogs: Evaluation of a bioabsorbable space-providing macroporous membrane with recombinant human bone morphogenetic protein-2. J. Periodontol..

[253] Meinel L., Fajardo R., Hofmann S., Langer R., Chen J., Snyder B., Vunjak-Novakovic G., Kaplan D. (2005). Silk implants for the healing of critical size bone defects. Bone.

[254] Kirker-Head C.A. (2000). Potential applications and delivery strategies for bone morphogenetic proteins. Adv. Drug Deliv. Rev..

[255] Taylor P., Allen S., Dreger S., Yacoub M. (2002). Human cardiac valve interstitial cells in collagen sponge: A biological three-dimensional matrix for tissue engineering. J. Heart Valve Dis..

[256] Wallace D.G., Rosenblatt J. (2003). Collagen gel systems for sustained delivery and tissue engineering. Adv. Drug Deliv. Rev..

[257] Kim C.S., Kim J.I., Kim J., Choi S.H., Chai J.K., Kim C.K., Cho K.S. (2005). Ectopic bone formation associated with recombinant human bone morphogenetic proteins-2 using absorbable collagen sponge and beta tricalcium phosphate as carriers. Biomaterials.

[258] Geiger M., Li R.H., Friess W. (2003). Collagen sponges for bone regeneration with rhBMP-2. Adv. Drug Deliv. Rev..

[259] Visser R., Arrabal P.M., Becerra J., Rinas U., Cifuentes M. (2009). The effect of an rhBMP-2 absorbable collagen sponge-targeted system on bone formation *in vivo*. Biomaterials.

[260] Wei G., Jin Q., Giannobile W.V., Ma P.X. (2007). The enhancement of osteogenesis by nano-fibrous scaffolds incorporating rhBMP-7 nanospheres. Biomaterials.

[261] MacNeil S. (2008). Biomaterials for tissue engineering of skin. Mater. Today.

[262] Angele P., Abke J., Kujat R., Faltermeier H., Schumann D., Nerlich M., Kinner B., Englert C., Ruszczak Z., Mehrl R. (2004). Influence of different collagen species on physico-chemical properties of crosslinked collagen matrices. Biomaterials.

[263] Sailer H.F. (1989). A new method of inserting endosseous implants in totally atrophic maxillae. J. Cranio-Maxillo-Fac. Surg..

[264] Ong J.L., Cardenas H.L., Cavin R., Carnes D.L. (1997). Osteoblast responses to BMP-2-treated titanium *in vitro*. Int. J. Oral Maxillofac. Implant..

[265] Piskounova S., Forsgren J., Brohede U., Engqvist H., Stromme M. (2009). *In vitro* characterization of bioactive titanium dioxide/hydroxyapatite surfaces functionalized with BMP-2. J. Biomed. Mater. Res. B Appl. Biomater..

[266] Bessho K., Carnes D.L., Cavin R., Chen H.Y., Ong J.L. (1999). BMP stimulation of bone response adjacent to titanium implants *in vivo*. Clin. Oral Implant. Res..

[267] Ishibe T., Goto T., Kodama T., Miyazaki T., Kobayashi S., Takahashi T. (2009). Bone formation on apatite-coated titanium with incorporated BMP-2/heparin *in vivo*. Oral Surg. Oral Med. Oral Pathol. Oral Radiol. Endod..

[268] Kim S.H., Park J.K., Hong K.S., Jung H.S., Seo Y.K. (2013). Immobilization of BMP-2 on a nano-hydroxyapatite-coated titanium surface using a chitosan calcium chelating agent. Int. J. Artif. Organs.

[269] Kim S.E., Yun Y.P., Lee J.Y., Shim J.S., Park K., Huh J.B. (2013). Co-delivery of platelet-derived growth factor (PDGF-BB) and bone morphogenic protein (BMP-2) coated onto heparinized titanium for improving osteoblast function and osteointegration. J. Tissue Eng. Regen. Med..

[270] Kim S.E., Kim C.S., Yun Y.P., Yang D.H., Park K., Kim S.E., Jeong C.M., Huh J.B. (2014). Improving osteoblast functions and bone formation upon BMP-2 immobilization on titanium modified with heparin. Carbohydr. Polym..

[271] Kim S.E., Song S.H., Yun Y.P., Choi B.J., Kwon I.K., Bae M.S., Moon H.J., Kwon Y.D. (2011). The effect of immobilization of heparin and bone morphogenic protein-2 (BMP-2) to titanium surfaces on inflammation and osteoblast function. Biomaterials.

[272] Lee S.C., Shea M., Battle M.A., Kozitza K., Ron E., Turek T., Schaub R.G., Hayes W.C. (1994). Healing of large segmental defects in rat femurs is aided by rhBMP-2 in PLGA matrix. J. Biomed. Mater. Res..

[273] Chen F., Wu Z., Wang Q., Wu H., Zhang Y., Nie X., Jin Y. (2005). Preparation and biological characteristics of recombinant human bone morphogenetic protein-2-loaded dextran-co-gelatin hydrogel microspheres, *in vitro* and *in vivo* studies. Pharmacology.

[274] Chen F., Wu Z., Jin Y., Wang Q., Du Y., Wang G. (2005). Preparation and property of recombinant human bone morphogenetic protein-2 loaded hydrogel nanospheres and their biological effects on the proliferation and differentiation of bone mesenchymal stem cells. Shanghai J. Stomatol..

[275] Chen F.-M., Zhao Y.-M., Sun H.-H., Jin T., Wang Q.-T., Zhou W., Wu Z.-F., Jin Y. (2007). Novel glycidyl methacrylated dextran (Dex-GMA)/gelatin hydrogel scaffolds containing microspheres loaded with bone morphogenetic proteins: Formulation and characteristics. J. Control. Release.

[276] Cao L., Wang J., Hou J., Xing W., Liu C. (2014). Vascularization and bone regeneration in a critical sized defect using 2-N,6-O-sulfated chitosan nanoparticles incorporating BMP-2. Biomaterials.

[277] Yang K., Cao W., Hao X., Xue X., Zhao J., Liu J., Zhao Y., Meng J., Sun B., Zhang J. (2013). Metallofullerene nanoparticles promote osteogenic differentiation of bone marrow stromal cells through BMP signaling pathway. Nanoscale.

[278] Wang Y., Mostafa N.Z., Hsu C.Y., Rose L., Kucharki C., Yan J., Jiang H., Uludag H. (2013). Modification of human bmsc with nanoparticles of polymeric biomaterials and plasmid DNA for BMP-2 secretion. J. Surg. Res..

[279] Kim T.H., Kim M., Eltohamy M., Yun Y.R., Jang J.H., Kim H.W. (2013). Efficacy of mesoporous silica nanoparticles in delivering BMP-2 plasmid DNA for *in vitro* osteogenic stimulation of mesenchymal stem cells. J. Biomed. Mater. Res. A.

[280] Park K.H., Kim H., Moon S., Na K. (2009). Bone morphogenic protein-2 (BMP-2) loaded nanoparticles mixed with human mesenchymal stem cell in fibrin hydrogel for bone tissue engineering. J. Biosci. Bioeng..

[281] Zhang S., Kucharski C., Doschak M.R., Sebald W., Uludag H. (2010). Polyethylenimine-PEG coated albumin nanoparticles for BMP-2 delivery. Biomaterials.

[282] Zhang S., Wang G., Lin X., Chatzinikolaidou M., Jennissen H.P., Laub M., Uludag H. (2008). Polyethylenimine-coated albumin nanoparticles for BMP-2 delivery. Biotechnol. Prog..

[283] Zhang S., Doschak M.R., Uludag H. (2009). Pharmacokinetics and bone formation by BMP-2 entrapped in polyethylenimine-coated albumin nanoparticles. Biomaterials.

[284] Kim K., Fisher J.P. (2007). Nanoparticle technology in bone tissue engineering. J. Drug Target.

[285] Cook S.D., Baffes G.C., Wolfe M.W., Sampath T.K., Rueger D.C. (1994). Recombinant human bone morphogenetic protein-7 induces healing in a canine long-bone segmental defect model. Clin. Orthop. Relat. Res..

[286] Cook S.D., Salkeld S.L., Rueger D.C. (1995). Evaluation of recombinant human osteogenic protein-1 (rhOP-1) placed with dental implants in fresh extraction sites. J. Oral Implantol..

[287] Sigurdsson T., Nygaard L., Tatakis D., Fu E., Turek T., Jin L., Wozney J., Wikesjö U. (1996). Periodontal repair in dogs: Evaluation of rhBMP-2 carriers. Int. J. Periodontics Restorative Dent..

[288] Sigurdsson T.J., Lee M.B., Kubota K., Turek T.J., Wozney J.M., Wikesjö U.M. (1995). Periodontal repair in dogs: Recombinant human bone morphogenetic protein-2 significantly enhances periodontal regeneration. J. Periodontol..

[289] Wikesjö U.M., Sorensen R.G., Kinoshita A., Jian Li X., Wozney J.M. (2004). Periodontal repair in dogs: Effect of recombinant human bone morphogenetic protein-12 (rhBMP-12) on regeneration of alveolar bone and periodontal attachment. J. Clin. Periodontol..

[290] Saito E., Saito A., Kawanami M. (2003). Favorable healing following space creation in rhBMP-2-induced periodontal regeneration of horizontal circumferential defects in dogs with experimental periodontitis. J. Periodontol..

[291] Jovanovic S.A., Hunt D.R., Bernard G.W., Spiekermann H., Wozney J.M., Wikesjö U.M. (2007). Bone reconstruction following implantation of rhBMP-2 and guided bone regeneration in canine alveolar ridge defects. Clin. Oral Implants Res..

[292] Selvig K.A., Sorensen R.G., Wozney J.M., Wikesjö U.M. (2002). Bone repair following recombinant human bone morphogenetic protein-2 stimulated periodontal regeneration. J. Periodontol..

[293] Urist M.R., Iwata H., Strates B.S. (1972). Bone morphogenetic protein and proteinase in the guinea pig. Clin. Orthop. Relat. Res..

[294] Hu W., Ye Y., Wang J., Zhang W., Chen A., Guo F. (2013). Bone morphogenetic proteins induce rabbit bone marrow-derived mesenchyme stem cells to differentiate into osteoblasts via BMP signals pathway. Artif. Cells Nanomed. Biotechnol..

[295] Spicer P.P., Kretlow J.D., Young S., Jansen J.A., Kasper F.K., Mikos A.G. (2012). Evaluation of bone regeneration using the rat critical size calvarial defect. Nat. Protoc..

[296] Turgeman G., Zilberman Y., Zhou S., Kelly P., Moutsatsos I.K., Kharode Y.P., Borella L.E., Bex F.J., Komm B.S., Bodine P.V. (2002). Systemically administered rhBMP-2 promotes msc activity and reverses bone and cartilage loss in osteopenic mice. J. Cell. Biochem..

[297] Marden L.J., Hollinger J.O., Chaudhari A., Turek T., Schaub R.G., Ron E. (1994). Recombinant human bone morphogenetic protein-2 is superior to demineralized bone matrix in repairing craniotomy defects in rats. J. Biomed. Mater. Res..

[298] Yasko A.W., Lane J.M., Fellinger E.J., Rosen V., Wozney J.M., Wang E.A. (1992). The healing of segmental bone defects, induced by recombinant human bone morphogenetic protein (rhBMP-2). A radiographic, histological, and biomechanical study in rats. J. Bone Jt. Surg. Am..

[299] Kirker-Head C.A., Gerhart T.N., Schelling S.H., Hennig G.E., Wang E., Holtrop M.E. (1995). Long-term healing of bone using recombinant human bone morphogenetic protein 2. Clin. Orthop. Relat. Res..

[300] Toriumi D.M., Kotler H.S., Luxenberg D.P., Holtrop M.E., Wang E.A. (1991). Mandibular reconstruction with a recombinant bone-inducing factor. Functional, histologic, and biomechanical evaluation. Arch. Otolaryngol. Head Neck Surg..

[301] Cook S.D., Baffes G.C., Wolfe M.W., Sampath T.K., Rueger D.C., Whitecloud T.S. (1994). The effect of recombinant human osteogenic protein-1 on healing of large segmental bone defects. J. Bone Jt. Surg. Am..

[302] Ripamonti U., van den Heever B., Sampath T.K., Tucker M.M., Rueger D.C., Reddi A.H. (1996). Complete regeneration of bone in the baboon by recombinant human osteogenic protein-1 (hOP-1, bone morphogenetic protein-7). Growth Factors.

[303] Margolin M.D., Cogan A.G., Taylor M., Buck D., McAllister T.N., Toth C., McAllister B.S. (1998). Maxillary sinus augmentation in the non-human primate: A comparative radiographic and histologic study between recombinant human osteogenic protein-1 and natural bone mineral. J. Periodontol..

[304] Den Boer F.C., Bramer J.A., Blokhuis T.J., van Soest E.J., Jenner J.M., Patka P., Bakker F.C., Burger E.H., Haarman H.J. (2002). Effect of recombinant human osteogenic protein-1 on the healing of a freshly closed diaphyseal fracture. Bone.

[305] Mizumoto Y., Moseley T., Drews M., Cooper V.N., Reddi A.H. (2003). Acceleration of regenerate ossification during distraction osteogenesis with recombinant human bone morphogenetic protein-7. J. Bone Jt. Surg. Am..

[306] Bostrom M.P., Saleh K.J., Einhorn T.A. (1999). Osteoinductive growth factors in preclinical fracture and long bone defects models. Orthop. Clin. N. Am..

[307] Bostrom M.P. (1998). Expression of bone morphogenetic proteins in fracture healing. Clin. Orthop. Relat. Res..

[308] Bax B.E., Wozney J.M., Ashhurst D.E. (1999). Bone morphogenetic protein-2 increases the rate of callus formation after fracture of the rabbit tibia. Calcif. Tissue Int..

[309] Herford A.S., Boyne P.J. (2008). Reconstruction of mandibular continuity defects with bone morphogenetic protein-2 (rhBMP-2). J. Oral Maxillofac. Surg..

[310] Sweeny L., Lancaster W.P., Dean N.R., Magnuson J.S., Carroll W.R., Louis P.J., Rosenthal E.L. (2012). Use of recombinant bone morphogenetic protein 2 in free flap reconstruction for osteonecrosis of the mandible. J. Oral Maxillofac. Surg..

[311] Govender S., Csimma C., Genant H.K., Valentin-Opran A., Amit Y., Arbel R., Aro H., Atar D., Bishay M., Borner M.G. (2002). Recombinant human bone morphogenetic protein-2 for treatment of open tibial fractures: A prospective, controlled, randomized study of four hundred and fifty patients. J. Bone Jt. Surg. Am..

[312] Tressler M.A., Richards J.E., Sofianos D., Comrie F.K., Kregor P.J., Obremskey W.T. (2011). Bone morphogenetic protein-2 compared to autologous iliac crest bone graft in the treatment of long bone nonunion. Orthopedics.

[313] Bibbo C., Patel D.V., Haskell M.D. (2009). Recombinant bone morphogenetic protein-2 (rhBMP-2) in high-risk ankle and hindfoot fusions. Foot Ankle Int..

[314] Moghaddam A., Elleser C., Biglari B., Wentzensen A., Zimmermann G. (2010). Clinical application of BMP 7 in long bone non-unions. Arch. Orthop. Trauma Surg..

[315] Dohin B., Fassier F., Hamdy R. (2009). Enhancement of difficult nonunion in children with osteogenic protein-1 (OP-1): Early experience. Clin. Orthop. Relat. Res..

[316] Moghaddam-Alvandi A., Zimmermann G., Büchler A., Elleser C., Biglari B., Grützner P., Wölfl C. (2012). Results of nonunion treatment with bone morphogenetic protein 7 (BMP-7). Unfallchirurg.

[317] Nicodemo A., Capella M., Deregibus M., Massè A. (2011). Nonunion of a sacral fracture refractory to bone grafting: Internal fixation and osteogenic protein-1 (BMP-7) application. Musculoskelet. Surg..

[318] Friedlaender G.E., Perry C.R., Cole J.D., Cook S.D., Cierny G., Muschler G.F., Zych G.A., Calhoun J.H., LaForte A.J., Yin S. (2001). Osteogenic protein-1 (bone morphogenetic protein-7) in the treatment of tibial nonunions. J. Bone Jt. Surg. Am..

[319] McKee M., Schemitsch E., Waddell J., Kreder H., Stephen D., Leighton R., Buckley R., Powell J., Wild L., Blachut P. The effect of human recombinant bone morphogenic protein (rhBMP-7) on the healing of open tibial shaft fractures: Results of a multi-center, prospective, randomized clinical trial. Proceedings of the 18th Annual Meeting of the Orthopaedic Trauma Association.

[320] Ristiniemi J., Flinkkilä T., Hyvönen P., Lakovaara M., Pakarinen H., Jalovaara P. (2007). rhBMP-7 accelerates the healing in distal tibial fractures treated by external fixation. J. Bone Jt. Surg. Br. Vol..

[321] Bilic R., Simic P., Jelic M., Stern-Padovan R., Dodig D., van Meerdervoort H.P., Martinovic S., Ivankovic D., Pecina M., Vukicevic S. (2006). Osteogenic protein-1 (BMP-7) accelerates healing of scaphoid non-union with proximal pole sclerosis. Int. Orthop..

[322] Bong M.R., Capla E.L., Egol K.A., Sorkin A.T., Distefano M., Buckle R., Chandler R.W., Koval K.J. (2005). Osteogenic protein-1 (bone morphogenic protein-7) combined with various adjuncts in the treatment of humeral diaphyseal nonunions. Bull. Hosp. Jt. Dis..

[323] McKee M., Schemitsch E., Waddell J., Wild L. The treatment of long bone nonunion with rhBMP: Results of a prospective pilot study. Proceedings of the American Academy of Orthopaedic Surgeons 71st Annual Meeting.

[324] Simmonds M.C., Brown J.V., Heirs M.K., Higgins J.P., Mannion R.J., Rodgers M.A., Stewart L.A. (2013). Safety and effectiveness of recombinant human bone morphogenetic protein-2 for spinal fusion: A meta-analysis of individual-participant data. Ann. Intern. Med..

[325] Fu R., Selph S., McDonagh M., Peterson K., Tiwari A., Chou R., Helfand M. (2013). Effectiveness and harms of recombinant human bone morphogenetic protein-2 in spine fusion: A systematic review and meta-analysis. Ann. Intern. Med..

[326] Carragee E.J., Hurwitz E.L., Weiner B.K. (2011). A critical review of recombinant human bone morphogenetic protein-2 trials in spinal surgery: Emerging safety concerns and lessons learned. Spine J..

[327] Hustedt J.W., Blizzard D.J. (2014). The controversy surrounding bone morphogenetic proteins in the spine: A review of current research. Yale J. Biol. Med..

[328] Carter S., Braem K., Lories R.J. (2012). The role of bone morphogenetic proteins in ankylosing spondylitis. Ther. Adv. Musculoskelet. Dis..

[329] Urist M.R. (1965). Bone: Formation by autoinduction. Science.

[330] Hadjiargyrou M., Lombardo F., Zhao S., Ahrens W., Joo J., Ahn H., Jurman M., White D.W., Rubin C.T. (2002). Transcriptional profiling of bone regeneration insight into the molecular complexity of wound repair. J. Biol. Chem..

[331] White A.P., Vaccaro A.R., Hall J.A., Whang P.G., Friel B.C., McKee M.D. (2007). Clinical applications of BMP-7/OP-1 in fractures, nonunions and spinal fusion. Int. Orthop..

